# Mild oxidative stress and dietary epigenetic modulators direct DNA methylation remodeling toward stress-resilience pathways

**DOI:** 10.1186/s12864-026-13088-w

**Published:** 2026-07-03

**Authors:** Javier A. Rodriguez-Casariego, Carmen Navarro-Guillén, Rubén Huesa-Cerdán, Juan Antonio Hidalgo-Pérez, Gonzalo Martínez-Rodríguez, Erick Perera

**Affiliations:** 1 Institute of Environment, Florida International University, Miami, FL USA; 2 Department of Marine Biology and Aquaculture, Andalusian Institute of Marine Sciences (ICMAN), Spanish National Research Council (CSIC), 11519 Cadiz, Puerto Real Spain

**Keywords:** Context-dependent epigenetic regulation, DNA methylation, Epigenetic remodeling, Gene regulatory networks, Nutritional epigenetics, Phenotypic plasticity, Redox signaling, Stress resilience

## Abstract

**Background:**

Although hepatic epigenetic plasticity in response to diet is well established, the factors that bias methylation remodeling toward adaptive gene networks remain unresolved. We hypothesized that mild oxidative stress acts as a contextual cue directing dietary epigenetic modulators toward DNA methylation remodeling of stress-resilience pathways. To evaluate this model, we analyzed hepatic transcriptional and methylation responses to a genistein plus vitamin C diet in seabream.

**Results:**

RNA-seq identified 146 differentially expressed genes (DEGs), with upregulated genes involved in SAM metabolism, chromatin remodeling, protein catabolism, and mitochondrial activity, and downregulated genes linked to glucose and lipid metabolism, intracellular trafficking, and signaling. Weighted gene co-expression network analysis revealed a modular transcriptomic organization associated with physiological traits, but only one module strongly correlated with diet. This module is related with metabolic activation and apoptosis control. Reduced representation bisulfite sequencing (RRBS) revealed 16,300 differentially methylated cytosines and 354 differentially methylated regions, primarily in gene bodies and intergenic regions. Functional Epigenetic Module analysis with remodeled promoters showed directed DNA methylation affecting epigenetic regulation, oxidative and metabolic stress, and stress adaptation, consistent with targeted remodeling. Functional in vivo validation revealed that the treatment reduces reactive oxygen species levels under oxidative stress.

**Conclusions:**

These findings demonstrate that induced mild oxidative stress combined with dietary epigenetic modulators produces a selective epigenetic remodeling of the liver. We propose that mild oxidative stress may act as a contextual signal that guides dietary epigenetic modulators to reshape DNA methylation landscapes in a pathway-directed manner.

**Supplementary Information:**

The online version contains supplementary material available at https://doi.org/10.1186/s12864-026-13088-w.

## Background

The regulation of oxidative stress is essential for maintaining health, wellbeing, growth, life-history trade-offs and overall fitness in animals. Oxidative stress arises when the production of reactive oxygen species (ROS) exceeds the capacity of the antioxidant defense system, resulting in cellular damage through the oxidation of lipids, proteins, nucleic acids, and organelles [1]. The liver is particularly vulnerable to oxidative damage due to its central role in metabolism, nutrient processing, and detoxification. In fish, both wild and farmed, oxidative stress can be triggered by multiple factors, including diet composition [2, 3], temperature variations [4–7], environmental contamination [8, 9], water quality [10], the use of anesthetics [11], osmotic challenges [12], food deprivation [13], hypoxia, stocking density, transport and handling among others [1, 14, 15]. Some dietary strategies have been shown to mitigate these effects such as the dietary supplementation with microbial additives, carotenoids, polysaccharides, polyphenols, vitamins, flavonoids, and synthetic antioxidants like butylated hydroxyanisole [16–20].

Most antioxidants act by scavenging free radicals or modulating redox-sensitive signaling pathways [21]. Beyond these direct effects, several studies in mammals have highlighted a complex interplay between oxidative stress and epigenetic regulation, particularly DNA methylation. ROS can alter DNA methylation patterns by depleting S-adenosylmethionine (SAM), the methyl donor used by DNA methyltransferases (DNMTs), or by affecting the activity of methionine adenosyltransferase (MAT), methionine synthase (MS) and ten eleven translocation (TET) enzymes [22]. Oxidative stress can also cause region-specific DNA hyper- or hypomethylation through the relocalization of DNMTs and alterations in chromatin structure and transcription factor binding [22]. These changes may produce transient [23] and long-lasting effects on gene expression [24]. These epigenetic effects may loop back to oxidative stress responses. Altered methylation in nuclear genes such as SOD2 or ZDHHC1 may increase oxidative stress [25, 26]. Promoter methylation of oxidative stress-related genes such as GPX and MnSOD, along with their upstream regulators KEAP1 and NRF2, has also been reported to affect stress responses [27]. At the hepatic level, diets deficient in methyl donors (e.g., methionine, choline) can induce non-alcoholic steatohepatitis (NASH)-like phenotypes, along with dysregulation of genes involved in redox metabolism [28]. Conversely, 1C-enriched (i.e., methionine, folic acid, and vitamins B6 and B12) diets improved redox capacity in Salmon muscle [29], and B12 supplementation enhanced antioxidant enzyme activity in zebrafish [30]. However, despite intense epigenetic research in fish [31], the functional relationships between their DNA methylation and regulation of oxidative stress responses remain largely unknown. Moreover, in both the livestock production and the biomedical science, there is a need to selectively manipulate the epigenome with loci, cell type, and temporal precision, outside the window of global reprogramming during early development [32].

The gilthead seabream (*Sparus aurata*) is a widely farmed marine fish. In this fish species, we previously showed that well-known antioxidants such as genistein (GEN) and vitamin C (VitC) [33, 34] when used at high doses in feed acts as slight pro-oxidants [35], as reported in other models [36, 37]. The levels of hepatic ROS and lipid peroxidation increased after feeding these supplements, but without signs of permanent damage [35]. Importantly, these compounds are also involved in epigenetic regulation. GEN exhibits DNA hypomethylating activity by inhibiting DNMTs [38, 39] and modulating the expression of DNMTs [40], while VitC promotes TET-mediated DNA demethylation [41, 42] and it may support the demethylating action of GEN as it does with other demethylating agents [41]. Indeed, both GEN [43] and VitC [44] have been related with the epigenetic modulation of Nrf2-mediated oxidative stress responses.

Given that DNA methylation remodeling is constrained by chromatin context, occurring preferentially in accessible genomic regions [32], we hypothesized that the combination of mild oxidative stress and dietary epigenetic modulators does not merely induce global methylation variability, but instead drives targeted DNA methylation remodeling at regulatory elements controlling stress-resilience pathways. Taking advantage of the dual properties of GEN and VitC, we fed juvenile seabream fish with a control and a GEN + VitC supplemented feed to generate the hepatic conditions to evaluate our hypothesis. Using liver samples from the feeding trial, we performed reduced representation bisulfite sequencing (RRBS) and RNA sequencing (RNA-seq) to profile genome-wide DNA methylation changes and gene expression. Our results provide novel insights into the epigenetic regulation of hepatic oxidative stress responses in fish and, more broadly, contribute to understanding how DNA methylation integrates redox regulation into liver physiology. Moreover, our findings support the development of strategies to direct the liver epigenome toward enhanced resilience.

## Results

### Diet-induced hepatic gene expression revealed changes in metabolism, tissue remodeling, and plasticity, and correlate with liver triglycerides

**Fig. 1:**
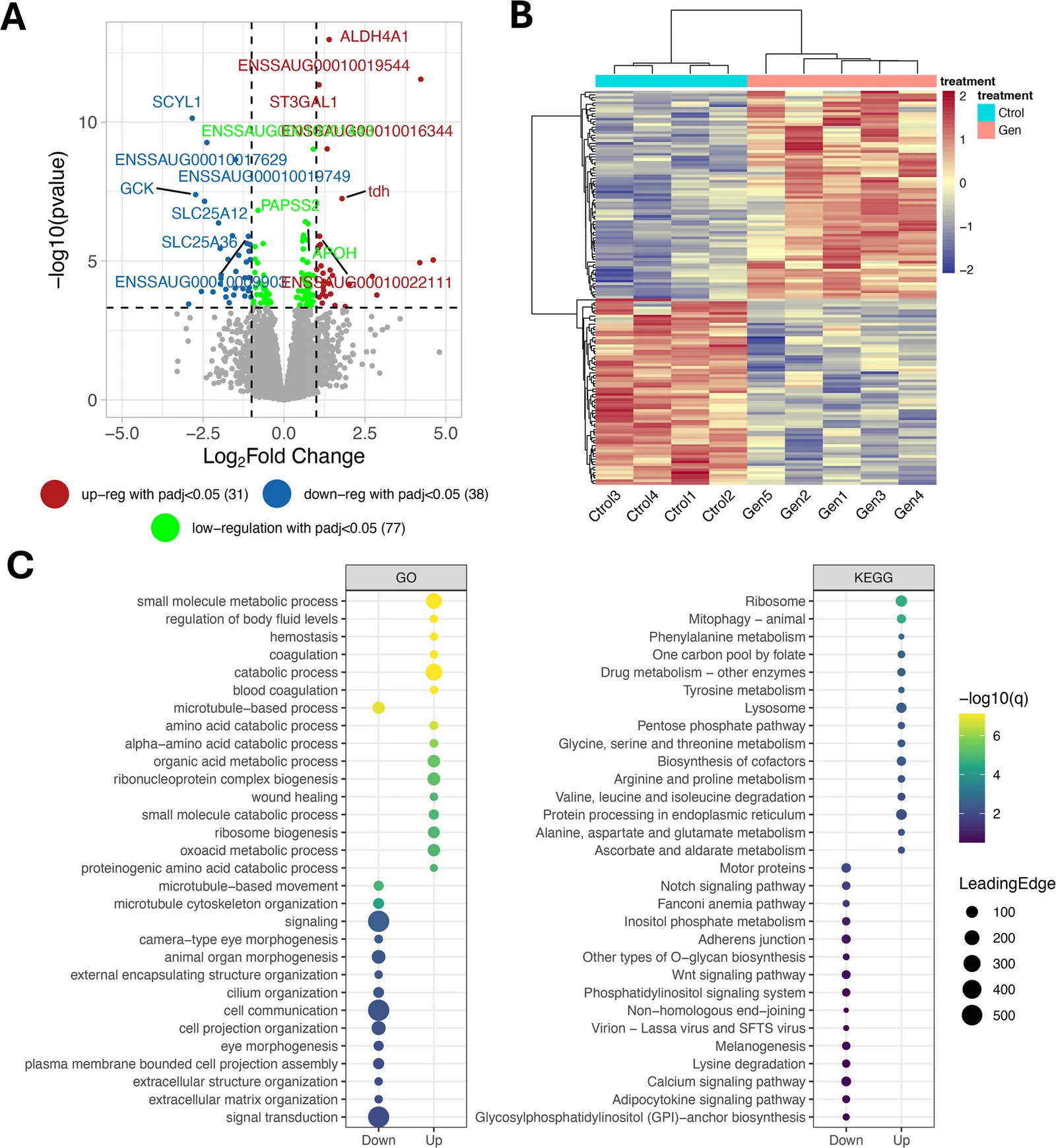
Transcriptional effects of GEN + VitC dietary supplementation in gilthead seabream. **A**. Volcano plot showing differential gene expression between fish fed the GEN + VitC–supplemented diet and control animals. Red and blue points denote significantly up- and downregulated genes (padj < 0.05), respectively, while green points represent genes with significantly reduced variability under supplementation. Several metabolic and stress-related genes with strong differential expression are labeled. **B**. Heatmap of all significantly differentially expressed genes, illustrating clear separation between dietary treatments and reduced inter-individual variability in supplemented fish. Rows represent genes and columns represent individual samples, with expression values scaled by gene. **C.** Functional enrichment analysis of Gene Ontology (GO) biological processes and KEGG pathways performed using Gene Set Enrichment Analysis (GSEA)

We performed RNA-seq analysis on liver samples from fish ingesting the control and the GEN + VitC diets. Between 97–98.6% of the 37.9–44.1 million raw reads obtained per sample passed quality filters and 78–84.7% were uniquely aligned to the reference genome, covering 19,229–20,360 genes (Additional file 1: Table ST1). Differential expression analysis identified 146 differentially expressed genes (DEGs; padj ≤ 0.05; Additional file 1: Table ST2), with 31 genes significantly up-regulated and 38 down-regulated with log2 fold change (|L2F|) > 1, and other 77 genes significantly affected (Fig. 1A). Upregulated genes with L2F > 1 included six un-annotated protein-coding genes, and genes involved in SAM synthesis (*mat1a*), chromatin remodeling (*rbbp4*), GABA degradation (*aldh5a1*), intracellular protein breakdown (cathepsin D-like, ENSSAUG00010016344), and amino-acid and carbohydrate metabolism (*aldh4a1*, *st3gal1*, and *tldh*) (Fig. 1A). Among down-regulated genes with L2F > −2 we obtained four un-annotated protein-coding genes, along with glucokinase (*gck*), the first step in most glucose metabolism pathways, a transcriptional regulator involved in hepatic intracellular trafficking (*scyl1*), and genes related with the metabolism of sterols and fatty acids (*cytochrome P450-like*, *scd1b*) and mitochondrial transport (*slc25a36*, *slc25a12*). Beyond these specific changes, the heatmap of DEGs reveals a clear shift in expression pattern, with clustering of samples according to experimental diets (Fig. 1B).

Gene set enrichment analysis (GSEA) of the differentially expressed genes revealed that the GEN + VitC diet induces an enrichment of genes related to metabolic and signaling pathways (Fig. 1C, Additional file 1: Table ST3). Upregulated pathways were dominated by small molecule metabolic process and catabolic process, including amino acid catabolism, among others such as ribosome related processes. In contrast, downregulated gene sets were enriched for extracellular matrix organization, cell communication, and signal transduction, including Notch and Wnt signaling pathways.

**Fig. 2:**
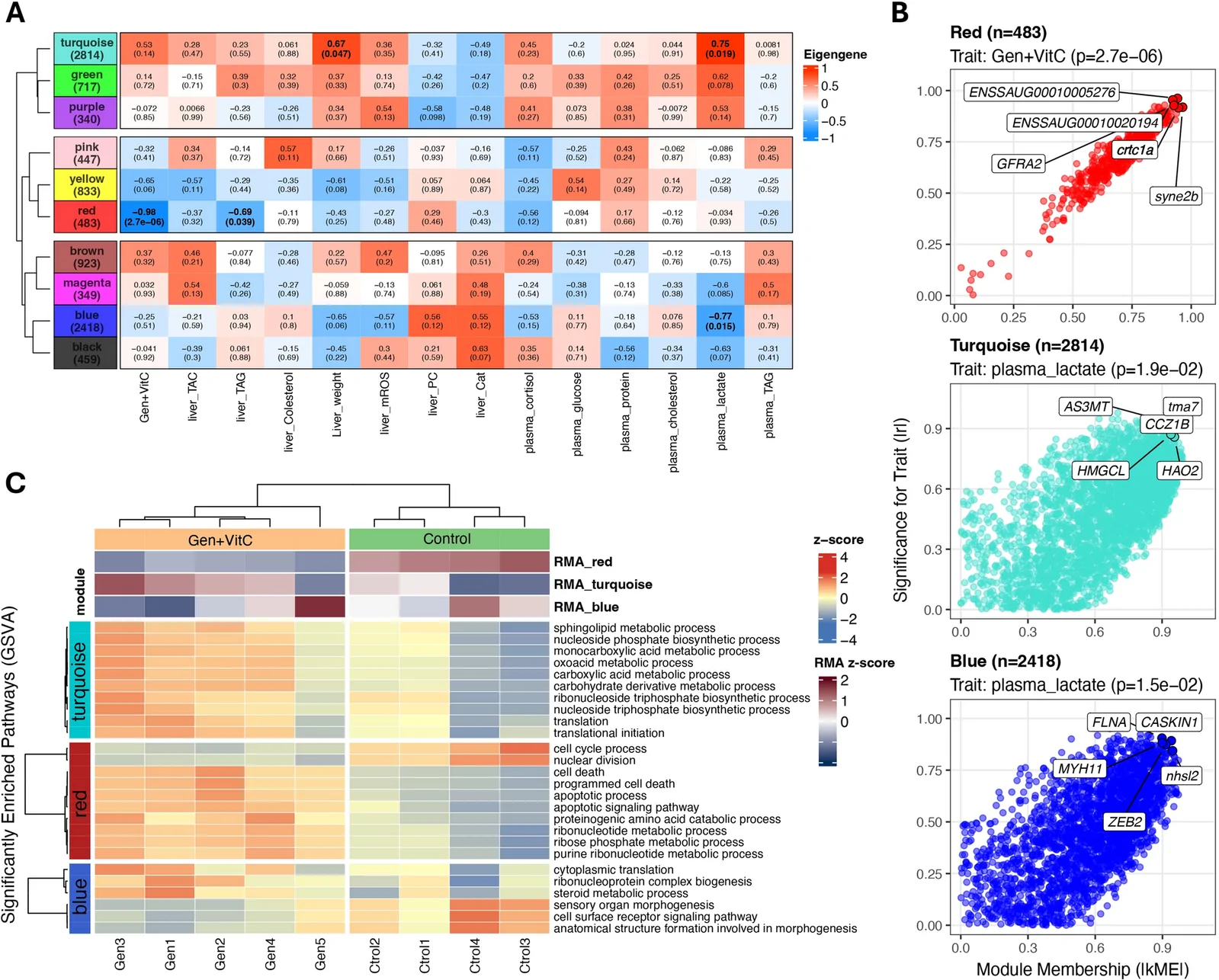
WGCNA gene co-expression network associated with Gen + VitC diet and physiological traits. **A**. Module–trait correlation heatmap for 10 WGCNA modules and the Gen + VitC treatment (coded 0 = control, 1 = Gen + VitC diet) together with liver and plasma physiological traits (liver weight, plasma lactate, liver catalase, plasma glucose, plasma triglycerides, liver phosphatidylcholine, plasma cortisol, liver triglycerides, liver total antioxidant capacity, liver mitochondrial ROS, plasma cholesterol, plasma protein, liver cholesterol). Each cell shows the Pearson correlation (top) and associated *p*-value (bottom; bold for *p* < 0.05) between the module eigengene and a given trait; colors indicate correlation strength (red = positive, blue = negative). The red, blue, and turquoise modules show trait significant associations. **B**. Relationships between gene significance for traits and absolute module membership (|kME|) values for the three trait-associated modules (red, blue, turquoise). Each point represents a gene within the module, with correlation FDR-corrected *p*-values indicated in each panel. Labeled points highlight representative hub genes with high |kME| and strong association with the diet. crtc1a is the hub gene of the red module. **C.** Heatmap of Gene Set Variation Analysis (GSVA) enrichment scores for significantly enriched GO Biological Process terms across all experimental samples (single sample GSEA; *q* < 0.05, |NES|≥ 1.8). Rows are grouped by their corresponding WGCNA module (Turquoise, Red, Blue), with the Relative Module Eigengenes Activity (RMA) for each module and treatment groups displayed in the top annotation. The main heatmap scale represents the relative pathway activity (z-score), where red indicates activation and blue indicates suppression. These results demonstrate that Gen + VitC supplementation shifts the liver transcriptome into distinct co-expression states, characterized by the upregulation of metabolic processes (Turquoise and Red) and the targeted suppression of cell cycle and signaling pathways (Red and Blue)

To understand the systemic transcriptomic response to GEN + VitC supplementation, weighted gene co-expression network analysis (WGCNA) was performed, identifying 10 distinct modules correlated with diet and physiological traits (Fig. 2). WGCNA network topology diagnostics is shown in Additional file 2: Fig. S1. The Red module (483 genes) exhibited a profound negative correlation with the GEN + VitC treatment (*r* = −0.98, *p* < 0.001), indicating widespread suppression of this gene network in supplemented fish (Fig. 2A). This module was also negatively correlated with liver TAG levels (*r* = −0.69, *p* = 0.039). Conversely, the Turquoise module (2,814 genes) demonstrated positive correlations with liver weight (*r* = 0.67, *p* = 0.047) and plasma lactate (*r* = 0.75, *p* = 0.019), while the opposite was observed for the Blue module (2,418 genes), negatively correlated with plasma lactate (*r* = −0.77, *p* = 0.015) and marginally with liver weight (*r* = −0.65, *p* = 0.06) (Fig. 2A).

To identify the core drivers within these highly correlated modules, we assessed the relationship between Module Membership (kME) and Gene Significance (*p-adjusted*) for the respective traits (Fig. 2B). The Red module displayed a robust correlation between kME and gene significance for the GEN + VitC treatment, revealing highly connected hub genes such as *syne2b*, *crtc1a,* and *gfra2*. Hub genes driving the Turquoise module's positive association with liver weight and plasma lactate included *hao2, hmgcl, tma7, ccz1b* and *as3mt*. The Blue module's negative association with plasma lactate was centrally driven by *flna*, *caskin1*, *myh11*, *nhsl2*, and *zeb2*.

To assess the functional consequences of these modular shifts, sample-specific pathway activity was quantified using Gene Set Variation Analysis (GSVA). Relative module activity (RMA) robustly distinguished Control from Gen + VitC samples across all three key modules (Fig. 2C). Gen + VitC supplementation induced a coordinated downregulation of pathways anchored in the Red module. This suppression was primarily driven by the attenuation of cell cycle processes, cell death, and apoptotic signaling pathways, alongside reductions in nucleotide and ribose phosphate metabolism. In stark contrast, the treatment sharply upregulated pathways within the Turquoise and Blue modules. The activated Turquoise network was highly enriched for metabolic turnover—specifically sphingolipid, monocarboxylic acid, and oxoacid metabolic processes—as well as translational initiation. Concurrently, the Blue module reflected heightened activation of cytoplasmic translation, ribonucleoprotein complex biogenesis, and developmental signaling, including sensory organ morphogenesis and cell surface receptor signaling pathways (Fig. 2C). Further integration of pathway-level activity with WGCNA module eigengenes is shown in Additional file 2: Fig. S2.

### Diet-induced remodeling of DNA methylation in regions related with the liver response to environmental or internal cues

**Fig. 3:**
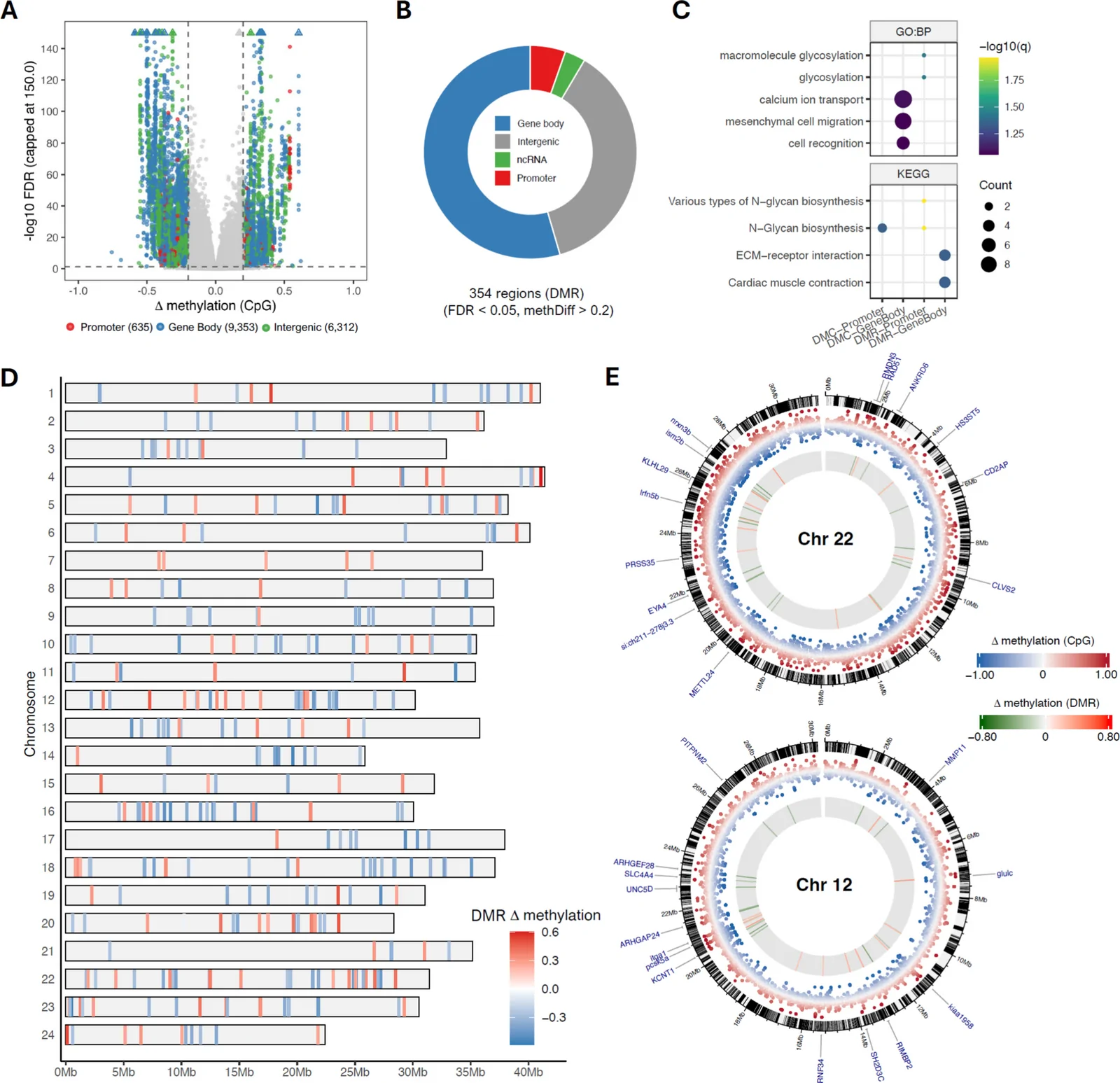
Genomic distribution and functional context of GEN + VitC–responsive DNA methylation changes in liver. **A**. Volcano plot of CpG methylation differences between GEN + VitC and control diets. The x-axis shows the difference in methylation proportion (Δ methylation, Gen + VitC – Control) for individual CpGs, and the y-axis shows –log10(FDR), capped at 150. Colored points highlight significantly differentially methylated CpGs/regions (FDR < 0.05) that pass effect-size thresholds, with grey points indicating non-significant sites. **B**. Donut plot showing the genomic distribution of differentially methylated regions (DMRs) across annotated features (e.g. promoters, exons, introns, intergenic), illustrating that most methylation changes occur in genic regions. **C.** GO biological process (top) and KEGG pathway (bottom) enrichment for genes overlapping DMRs. Dot color indicates enrichment strength (–log10 *q*) and dot size the number of DMR-associated genes in each term. DMRs are preferentially associated with genes involved in macromolecule glycosylation, calcium ion transport, mesenchymal cell migration and recognition, as well as N-glycan biosynthesis and ECM–receptor interaction. **D**. Chromosomal ideogram showing the physical distribution of DMRs across the 24 chromosomes. Each bar represents a chromosome, with vertical segments marking DMR positions and colored by the direction and magnitude of Δ methylation (red = hypermethylated in GEN + VitC; blue = hypomethylated). **E**. Circos plots summarizing DMRs and their associated regions. The outer track shows chromosomes; inner tracks show DMRs colored by Δ methylation CpG methylation levels

Given the observed restructuring of hepatic co-expression networks by the GEN + VitC diet, we next asked whether restructuring of DNA methylation occur in response to GEN + VitC supplementation, potentially contributing to the regulation of these transcriptional responses. After RRBS analyses, we obtained between 35.4 to 47.7 M reads per sample, with 62.2–64.9% of reads uniquely aligned to the reference genome, and covering 4.8–5.9 M of unique CpGs (Additional file 1: Table ST4). DNA methylation levels (> 70% in gene bodies) were the lowest around transcription start sites and CpG islands, with only promoter (−2 kb to + 200 bp from TSS) methylation showing a general negative correlation with expression (Additional file 2: Fig. S3). The dietary intervention produced DNA methylation remodeling in 16,300 CpGs (DMCs, fdr < 0.05 and 15% Δ meth) and 354 regions (DMRs, fdr < 0.05 and > 20% Δ meth) (Fig. 3A, B, Additional file 1: Table ST5). Most DMRs occurred at gene bodies and intergenic regions (Fig. 3B), with both hypo- and hypermethylated DMRs overlapping all genomic regions, although the number of hypomethylated DMRs was higher (Additional file 2: Fig. S4) as expected by the demethylating nature of GEN and VitC. Functional analyses revealed enrichment of diet-induced DNA methylation changes in processes related with calcium transport, cell recognition and migration, and ECM (extracellular matrix)—receptor interaction (Fig. 3C, Additional file 1: Table ST6). DMRs were distributed across all chromosomes (Fig. 3D), with chromosomes 12 and 22 exhibiting the highest density of DMRs overlapping genes (Fig. 3E). Several gene promoters (−2 kb to + 200 bp from TSS) were also found to be enriched by DMCs (Additional file 2: Fig. S5) with more consistent changes in DNA methylation occurring in the promoters of *acadsb*, *ppm1nb*, *rtn2b*, *nr0b1*, and *sash1*, involved in metabolism of branched-chain amino acids and fatty acids, regulation of Wnt signaling, glucose uptake, negative regulation of transcription, and TLR4 signaling, respectively. In addition, a longer list of genes contained numerous DMC in extended regions (gene body + −2 kb to + 200 bp promoter) (Additional file 2: Fig. S6). Curiously, a clear switch in DNA methylation was observed for a cluster containing several genes with roles in cell organization and signaling (*arhgef49*, *fmnl1*, *tmem8b*, *cldn11*, *sh2d3c*, *cacna2d1a*, *nat8*, *ikzf5*, *znf385c*), and some DEGs were also represented among the DMC enriched genes (Additional file 2: Fig. S6).

### Gene expression changes associated with DNA methylation remodeling mostly affect hepatic epigenetic regulation, stress adaptation, and redox balance

**Fig. 4:**
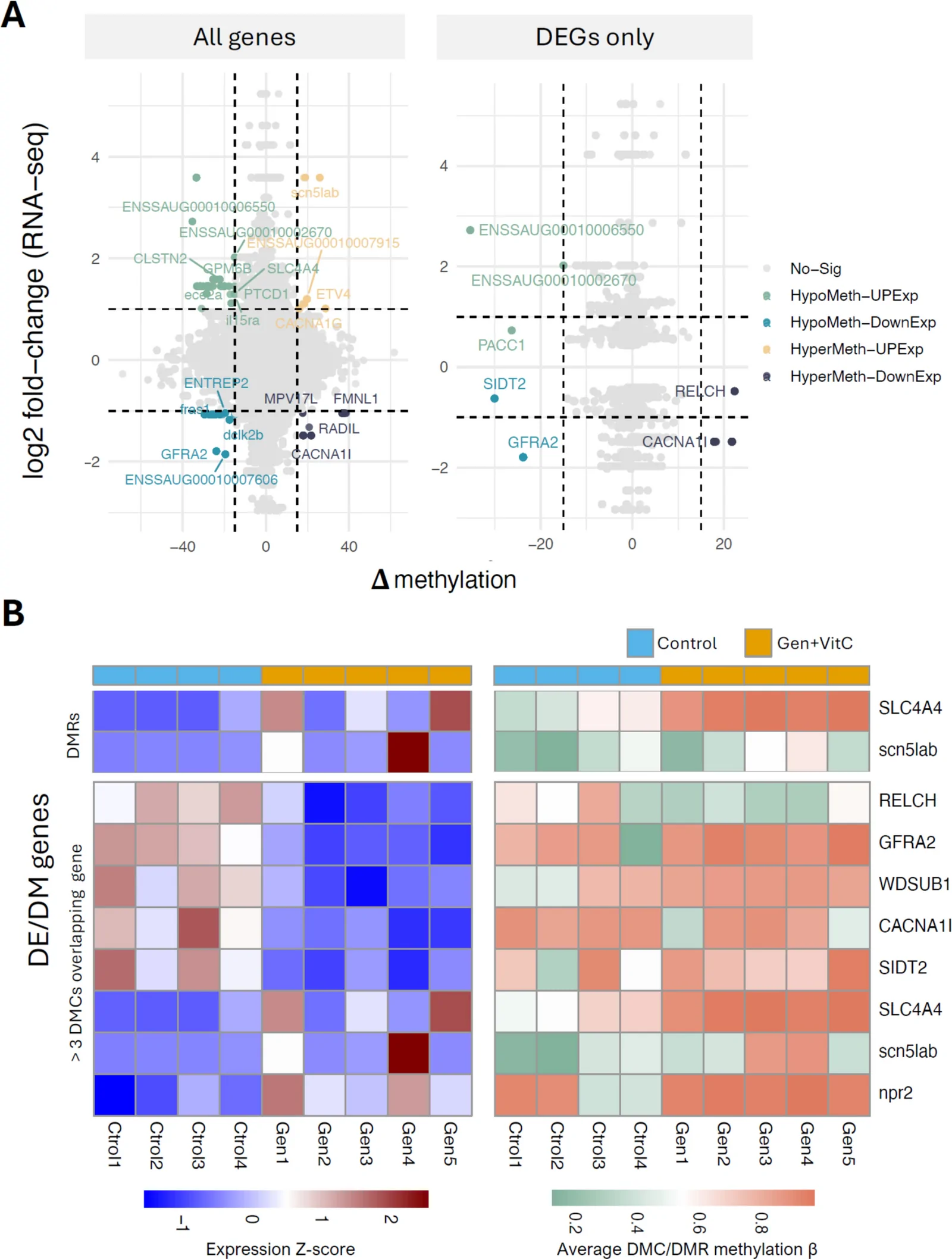
Integration of DNA methylation and gene expression responses to the GEN + VitC diet. **A**. Relationship between gene-level DNA methylation changes and transcriptional responses in liver. Each point represents a gene with both methylation and expression data, plotted as change in CpG methylation (Gen + VitC – control) versus log₂ fold-change in expression. The left panel shows all genes with DMC (> 5) overlaps, whereas the right panel shows differentially expressed genes (DEGs) only. Points are colored according to four methylation–expression categories (e.g. hypermethylated & upregulated, hypermethylated & downregulated, hypomethylated & upregulated, hypomethylated & downregulated), and representative genes with large and/or concordant changes are labeled. **B**. Heatmaps summarizing mean methylation (left) and expression (right) profiles for genes in each methylation–expression category across individual samples (control and GEN + VitC fish indicated by colored bars above the heatmaps). Colors in the methylation heatmaps represent relative gain (red) or loss (blue) of methylation in supplemented fish, whereas colors in the expression heatmaps represent relative up- (red) or down-regulation (green) in GEN + VitC fish

**Fig. 5:**
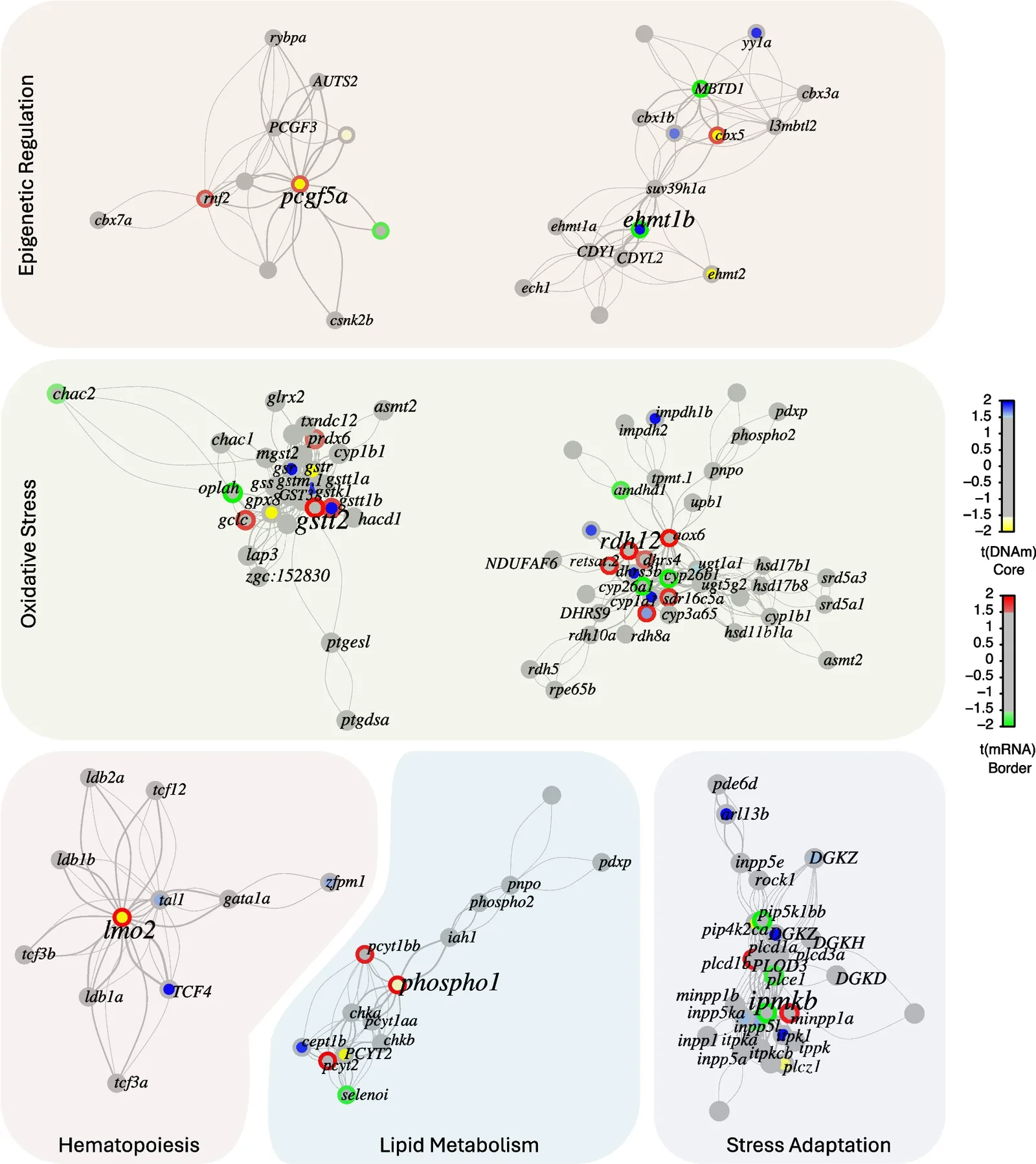
Results of functional epigenetic modules (FEM) analysis for promoter methylation. Representative interactomes linked to 5 functional categories (epigenetic regulation, oxidative stress, hematopoiesis, lipid metabolism and stress adaptation) are shown centered around their hub gene. DNA methylation change (yellow to blue scale) is represented as the core color of each node, and gene expression change (green to red scale) is represented as the border. Networks connectors are based on protein–protein interaction (PPI) networks

Regardless of the evidence of substantial remodeling at the transcriptional and DNA methylation levels, direct overlap between DMC/DMRs and DEGs was observed only for a few genes (Fig. 4). Hypermethylation was associated with significant downregulation of *relch* and *cacna1ia*, while hypomethylation with downregulation of *gfra2* and *sidt2*, and upregulation of *pacc1*, a *pcdhac2*-like gene (ENSSAUG00010002670), and a non-annotated gene (Fig. 4). Thus, we further evaluated the relationship between diet-induced changes in DNA methylation and gene expression in the context of protein–protein interaction networks using functional epigenetic modules (FEM) analysis based on promoter methylation. Seven interactomes hotspots were retrieved (Fig. 5; Additional file 1: Table ST7) with seeds at *ehmt1b*, *pcgf*5, *gstt2*, *rdh12*, *ipmkb*, *phospho1* and *lmo2*. Clear relationships were found between promoter hypomethylation and increased expression in *cbx5*, *pcgf5*, *phospho1*, and *imo2*, while between promoter hypermethylation and downregulation of expression of *ehmt1b*. Other relationships were also observed such as hypermethylation and increased expression of *gstt1b*. Several changes in DNA methylation within modules were not correlated with changes in gene expression at a single gene level, but with changes in gene expression of functionally related genes. Overall, modules can be classified as epigenetic regulation modules (*ehmt1b* and *pcgf5*), oxidative and metabolic stress response modules (*gstt2* and *rdh1*), stress adaptation module (*ipmkb*), phospholipid metabolism module (*phospho1*), and hematopoiesis module (*lmo2*).

### Treatment modulates reactive oxygen species levels under oxidative stress in vivo

**Fig. 6:**
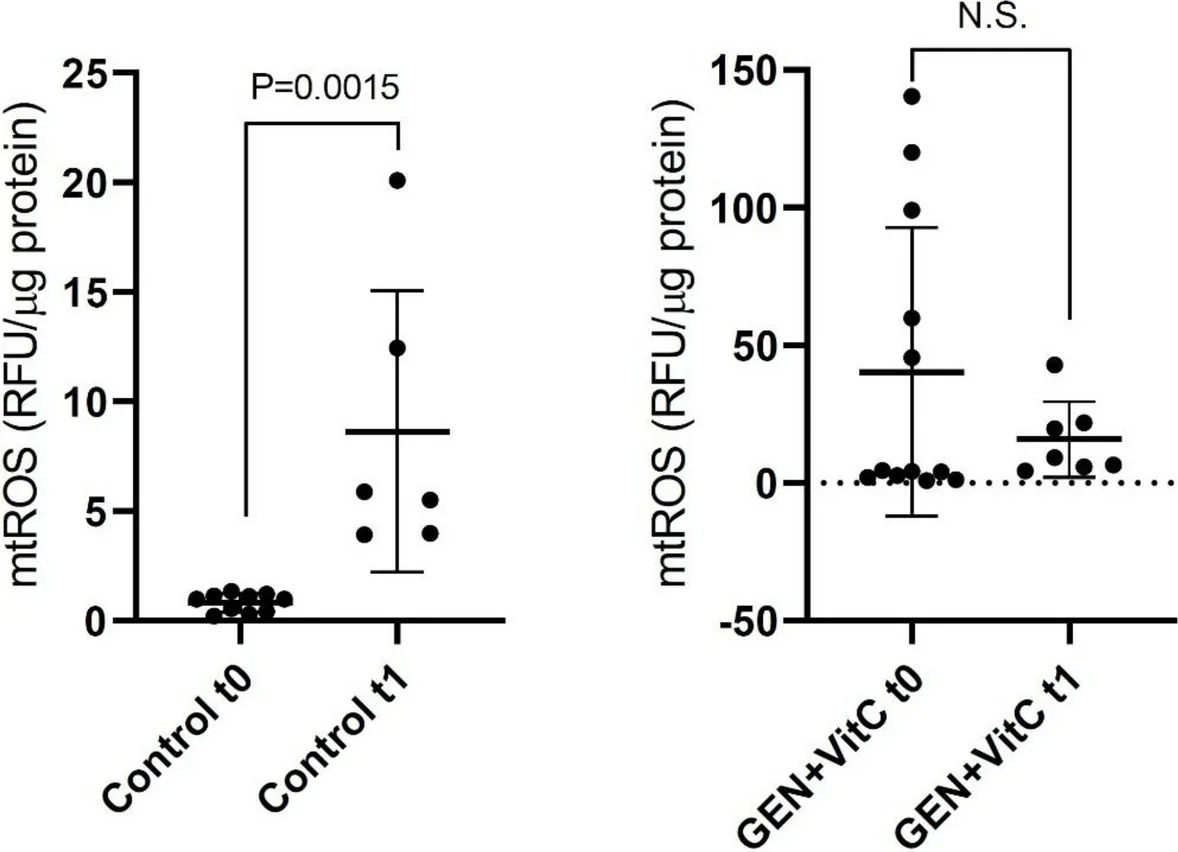
Oxidative stress (mitochondrial ROS production, mtROS) in liver of gilthead seabream under a control or GEN + VitC supplemented diet before (t0) and after (t1) an air exposure challenge. Twelve fish per dietary group were sampled at t0 and seven fish per group at t1. Outliers were identified using the ROUT method (Q = 1%), yielding final sample sizes of *n* = 10 (Control t0), *n* = 12 (GEN + VitC t0), *n* = 6 (Control t1), and *n* = 7 (GEN + VitC t1). t-test results are shown. Significance was set at *p* < 0.05

We exposed these two groups of fish to a three minutes air exposure and one-hour recovery challenge. This is known to produce hepatic oxidative stress due to rapid reoxygenation during recovery that favor a burst in mtROS production. We observed that while control fish exhibited the expected increase in hepatic mtROS production during recovery, the GEN + VitC fish did not exhibited this effect (Fig. 6).

## Discussion

### Diet-induced restructuring of hepatic gene networks associated with physiological traits

The transcriptomic results reveal a clear shift in expression pattern due to dietary manipulation, with predicted changes in metabolic and signaling pathways. Upregulated pathways were dominated by amino-acid catabolism, one-carbon/folate metabolism, pentose-phosphate activity, and lysosomal processing, consistent with enhanced redox capacity [45–47]. In contrast, downregulated gene sets were enriched for cytoskeletal organization, extracellular matrix remodeling, and developmental signaling pathways such as *Wnt* and *Notch*, indicating suppression of energy-intensive structural and proliferative processes. Together, the GSEA results highlight a coordinated shift from proliferative and structural programs toward a metabolically centered state in supplemented fish, suggesting an adaptive metabolic rewriting [48].

Weighted gene co-expression network identified different modules, and some of them correlated with physiological traits. Two identified modules exhibited opposite relationships with liver weight and plasma lactate, but not impacted by diet, indicating that distinct transcriptional programs contribute to variation in hepatic mass. However, only one module strongly correlated with the Gen + VitC treatment and liver triglyceride content, with strong relationships between hub-gene status and diet. This pattern shows that genes most central to this module are also those most strongly associated with the dietary manipulation, highlighting candidate hub genes that may coordinate the transcriptional response to Gen + VitC. The identification of *syne2*, *crtc1*, and *gfra2* as hub genes associated with diet and hepatic triglyceride content supports a coordinated response linking metabolic regulation, cellular architecture, and signaling pathways in the liver. Further functional analysis supports that GEN + VitC supplementation coordinates a systemic transcriptomic shift, suppressing specific morphogenic and structural networks while upregulating primary energetic and catabolic pathways.

Modular transcriptomic responses to different kind of stress (e.g. heat stress, hypoxia) have been reported before in fish [49, 50]. However, WGCNA results show that the GEN + VitC diet does not induce global transcriptomic changes but rather mostly affects a specific gene module associated with a key physiological readout of metabolic state, along with cell cycle processes, cell death, and apoptotic signaling pathways. While these relationships are correlative, they provide a mechanistic framework for future functional work targeting specific hub genes and pathways.

### Selective DNA methylation remodeling toward stress-resilience pathways

This work revealed that the dietary intervention with GEN + VitC produced extensive DNA methylation remodeling of the liver. Functional analyses revealed enrichment of diet-induced DNA methylation changes in processes related with calcium transport, cell recognition and migration, and extracellular matrix receptor interactions, suggesting that these changes may be involved in modulating the liver’s responsiveness to environmental or internal clues. Overall, DNA methylation results are functionally in line with observed transcriptional responses. For example, hypermethylation was associated with significant downregulation of a gene involved in intracellular cholesterol distribution (*relch*) [51] and Ca^2+^-signaling in the liver (*cacna1ia*) [52]. On the other hand, hypomethylation was found to be associated with downregulation of a receptor for growth factors (*gfra2*) [53] and a gene involved in RNA degradation (*sidt2*) [54]. In the liver, *gfra2* is enriched in Kupffer cells [55] and it has been related with the feeding processes though with varying results. Probiotic administration upregulated *gfra2* in the liver of the olive flounder *P. olivaceus* [56], GFRA2 knock-out mice are unable to digest food correctly [57], and in beef cattle, increased hepatic expression of GFRA2 correlated with improved feed efficiency [58], although no differences were found between fish ingesting the control or the GEN + VitC diets regarding feed intake, growth rate or feed efficiency [35]. Finally, a relationship between hypomethylation and gene upregulation was evident for a gene involved in acid-induced cell death (*pacc1*), and a cell-adhesion protein (*pcdhac2*-like).

However, as direct overlap between DMC/DMRs and DEGs was observed only for a few genes, which is consistent with extensive evidence supporting that direct methylation/expression relationships are elusive for most non-mammalian models, we evaluated the relationship between diet-induced changes in DNA methylation and gene expression using functional epigenetic modules (FEM) analysis.

Changes in two modules (*ehmt1b* and *pcgf5*) revealed changes in networks involved in the epigenetic regulation of gene expression. In the *ehmt1b* module, the repressive *cbx5* was up-regulated. This gene encodes for a chromatin reader of H3K9me3 marks, involved in the formation and maintenance of heterochromatin [59]. In contrast, *ehmt1b*, which encodes a histone methyltransferase generally repressing gene expression by adding H3K9me1-2 [60], was down-regulated. As the product of EHMT1 is needed for CBX5 to bind, this opposed DNA methylation-driven expression patterns, suggest a selective remodeling of hepatocytes rather than a global silencing. Finally, *mbtd1*, a chromatin reader that promotes gene activation but also regulate bivalent chromatin as component of the TIP60 chromatin remodeling complex [61], was down-regulated. Likewise, in the *pcgf5* module, two genes (*rnf2* y *pcgf5*) with roles in the epigenetic repression of gene expression through the Polycomb Repressive Complex 1 (PRC1) were upregulated. Overall, the activation of chromatin repressors (*cbx5, rnf2, pcgf5*) alongside suppression of activators (*mbtd1*) suggest a shift in epigenetic regulatory balance toward a more repressive chromatin state.

Two modules revealed coordinated responses to oxidative and metabolic stress. In a clear oxidative stress/detoxification response module (*gstt2*), we observed upregulation of genes whose encoded proteins directly reduce peroxides and lipid hydroperoxides such as *prdx6*, that produces (*gclc*) the rate-limiting enzyme in the synthesis of the main intracellular antioxidant, glutathione, or that enhanced glutathione-based detoxification (*gstt1b*, *gstt2*). The concomitant downregulation of *oplah* indicated that the nutritional intervention induced a shift from the recycling of glutamate via *oplah* to de novo synthesis via *gclc*, probably due to higher requirements of glutamate under mild oxidative stress [62]. In the related module (*rdh12*), we found upregulation of genes that collectively contribute to enhanced retinoid recycling and retinal detoxification (*rdh12*, *dhrs4*, *sdr16c5a*, *aox6*) [63] and suppressed retinoic acid (RA) degradation *(cyp26a1/b1*) [64], likely resulting in higher levels of RA, a potent transcriptional regulator of genes related with development [65] and metabolism [66]. Current results collectively indicate that mild oxidative stress produced by the nutritional intervention [35] led to integrated redox and metabolic defense responses, in which DNA methylation remodeling plays a role. Analyses of molecular functions affected in zebrafish F1 offspring by parental 1C-micronutrient deficiency also revealed changes in the cellular response to oxidative and endoplasmic reticulum (ER) stress [67].

The module *ipmkb* represents a shift away from proliferative and anabolic signaling toward stress adaptation. It suggests a decrease of inositol phosphate and phosphoinositide signaling due to downregulation of *ipmkb* and *pip5klb*b, which may decrease AKT/mTOR activity and growth signals [68], and ER calcium retention via downregulation of *plce1*, which may avoid oxidative damage [69]. The last module (*phospho1*) points to changes in phospholipid metabolism. It exhibited upregulation of *pcyt1bb* and *pcyt2*, related with the synthesis of phosphatidylcholine (PC) and phosphatidylethanolamine (PE), respectively [70]. The concomitantly upregulation of *phospho1*, which hydrolyze phospholipids [71], suggests an increase in phospholipid turnover, rather than net synthesis. Indeed, downregulation of *selenoi* would indicate a decrease at the final step of PE synthesis [72]. These responses may oppose oxidative membrane damage, given that animals from this group exhibited higher level of lipid peroxidation respect to controls, although protein carbonylation remain unaffected [35]. Lipid peroxidation occurs at early phases of oxidative stress and protein carbonylation later, when endogenous antioxidant defenses are insufficient to neutralize the buildup of ROS and other reactive species [73]. This confirms that DNA methylation remodeling occurred under mild oxidative stress induced by high doses of GEN + VitC, preceding detectable signs of cellular damage. We showed that this cellular environment triggered adaptive cellular responses and may directed DNA methylation remodeling toward functionally relevant genomic regions. In our previous study on seabream liver explants, treatment with the DNA methylation inhibitor decitabine did not induce oxidative stress during 48 h of culture [74], and FEM analysis of DNA methylation changes revealed interactomes associated with protein trafficking and signal transduction [75], but not with oxidative or metabolic stress responses, or with epigenetic remodeling.

### Functional validation of hepatic stress resilience

As transcriptional responses to moderate hepatic oxidative stress were accompanied by directed DNA methylation remodeling, they may represent more than a transient response, potentially indicating a shift toward a new functional state. This hormetic-like effect may confer increased resilience to further oxidative challenges. To examine this issue, we exposed these two groups of fish to a three minutes air exposure and one-hour recovery challenge. This is known to produce hepatic oxidative stress due to rapid reoxygenation during recovery that favor a burst in ROS production. We observed that while control fish exhibited the expected increase in hepatic ROS production during recovery, the GEN + VitC fish did not exhibited this effect, which indicates a faster oxidative stress response. While we demonstrated a clear increase in hepatic stress resilience in supplemented fish, the mechanism remains to be stablished and, regarding DNA methylation remodeling, evidence for causality still need to be produced. There is limited evidence of the fitness advantage of epigenetic remodeling under preconditioning in both vertebrates [76] and invertebrates [77]. Conversely, it is known that the liver of a mouse model of chronic oxidative stress is protected against fatty liver injuries [78] which is thought to results from DNA methylation remodeling [79]. Also, the Nrf2‐mediated oxidative stress response pathway was the most abundant pathway associated with enhancers that overlapped with VitC ‐increased 5‐hmC peaks in bladder cancer [44]. However, unlike studies on chronic stress or pathological conditions that induced maladaptive epigenetic remodeling, our results suggest that mild oxidative stress is associated with a more selective reorganization of DNA methylation at stress-responsive genomic regions.

Several results in other fish species [67, 80–83] and our previous work in seabream [35] pointed to different beneficial effects on fish physiology and behavior of maintaining the methylation potential through nutrition (e.g. 1 C nutrients). However, even keeping appropriated levels of 1 C nutrients, this study reveals that a dietary manipulation with modulators of DNA demethylation may prepare fish to cope with hepatic stressors. Further studies will be necessary to determine whether these findings extend to other contexts (e.g. lipogenic diets, heat waves), and whether the observed epigenetic changes confer long-term resilience by maintaining relevant genomic regions in a poised state. In this regard, while epigenetic poising and epigenetic memory have been extensively described in plants and in mammalian systems such as innate immunity and stress-related pathways, their potential role in the liver of vertebrates remains largely unexplored.

## Conclusions

This study has some limitations that warrant recognition. Although mild oxidative stress was induced through diet and this condition is known to target DNA methylation remodeling, the nutrients responsible for this effect also have recognized epigenetic activities. Therefore, the extent to which GEN and VitC contribute to potentiating ROS-induced epigenome remodeling through their actions on DNMTs and TETs remains to be quantitatively determined. In addition, the discrete nature of diet compared to the continuous oxidative stress responses may reduce statistical power and hinder the detection of a higher number of significant correlations between DNA methylation and stress responses.

Nevertheless, although fish responses to liver stress have been previously studied, including metabolic adjustments, ER stress, and ROS scavenging responses [84, and references therein], this study highlights the coordinated associations between DNA methylation remodeling and oxidative stress responses in fish, also supporting the idea that these processes may be modulated through nutriepigenomic interventions. In this context, the present study shows that under mild oxidative stress and in the presence of dietary epigenetic modulators, DNA methylation remodeling primarily affects hepatic redox balance, lipid metabolism, signaling pathways, and epigenetic regulation, thereby providing insights into how methylome remodeling can be partially targeted to functionally relevant genomic regions. Thus, we propose that mild oxidative stress may act as a contextual signal that guides dietary epigenetic modulators to reshape DNA methylation landscapes in a pathway-directed manner.

## Methods

### Source of the animals and ethics

Fish were obtained from a commercial source, Valenciana de Acuicultura (VASA), and kept at the registered facilities (REGA: ES110280000311) of the Instituto de Ciencias Marinas de Andalucía (ICMAN-CSIC). The experiment followed the Guidelines of the Spanish (RD53/2013) and European Union Council (2010/63/EU) for the use and experimentation of laboratory animals and was approved by the Spanish National Research Council (CSIC) Bioethical Committee and the Autonomous Andalusian Government (authorization reference no. 24/10/2023/092). This species is a protandrous hermaphrodite. Individuals mature first as males, and a proportion later change sex to female. All sampled individuals were juveniles and thus males.

### Feeding trial and liver samples

Liver samples (*n* = 5 per treatment) analyzed in this work were collected in DNA/RNA Shield (Zymo Research) in our previous experiment [35], where we fed juveniles of the gilthead seabream with a control (analogous to a commercial diet) and with 1C-micronutrients and genistein/vitamin C (GEN + VitC) enriched feeds for 2 months. Fish were manually fed with the experimental diets three times per day (8:00 h, 12:30 h, and 15:00 h) until apparent satiation (ad libitum). Extensive details on the experimental design, culture conditions, diet composition, and sampling methods were provided in our previous work [35]. After intense phenotyping, different short- and long-term effects were found after these nutritional interventions. However, within the liver, differences were only found in the levels of oxidative stress and between the control and GEN diet; mitochondrial ROS production and lipid peroxidation were significantly higher in fish from the GEN + VitC group respect to controls [35]. Therefore, only liver samples from these two groups were analyzed here. In particular, the GEN + VitC diet was similar to the control diet but supplemented with GEN at 1.5 g kg^−1^ as a DNA methylation inhibitor, and also with VitC at 3 g kg^−1^ to potentiate the action of GEN [41, 42].

### DNA/RNA extraction

Whole tissue samples were submitted to Zymo Research (Irvine, CA) for both RNA-seq and RRBS analyses. The Quick-DNA/RNA Plus Microprep Kit was used to extract RNA and DNA simultaneously. The quality and integrity of RNA and DNA were verified using an Agilent TapeStation. Cutoff for RNA integrity was RIN ≥ 8.5.

### RNA-Seq Library Preparation and Sequencing

RNA libraries were generated from total RNA using the Zymo-Seq RiboFree Total RNA Library Prep Kit, following the manufacturer’s instructions. Library quality and size distribution were assessed using the Agilent D1000 ScreenTape Assay on a TapeStation. Sequencing was performed on an Illumina NovaSeq platform, producing 150 bp paired-end reads with a minimum depth of 30 million read pairs per sample.

### RNA-Seq data processing and analysis

Bioinformatic processing was conducted using a modified version of the Zymo Research RNA-Seq pipeline (v3.0.0). Initial quality assessment of raw reads was performed with FastQC v0.11.9 (http://www.bioinformatics.babraham.ac.uk/projects/fastqc). Adapters and low-quality bases were removed using Trim Galore! v0.6.7 [85]. Cleaned reads were aligned to the *S. aurata* reference genome (fSpaAur1.1; GCF_900880675.1) using STAR v2.6.1d [86] with parameters from the nf-core/RNAseq pipeline [87]. Alignment files were filtered and indexed with SAMtools v1.14 [88]. Quality metrics for sequencing and alignment were evaluated using RSeQC [89] and QualiMap [90]. Duplicate reads were identified and flagged using Picard MarkDuplicates v2.26.3 (http://broadinstitute.github.io/picard/), while library complexity was estimated via Preseq v2.0.3 [91]. Duplication statistics were assessed with dupRadar v1.22.0 [92]. Gene-level read counts were generated using featureCounts v2.0.1 [93].

Genes with a mean count below 1 were excluded, and the remaining counts were normalized using the variance stabilizing transformation (VST) in DESeq2 v1.32.0 [94], and differential expression was computed with DESeq2. To enable improved pathway analysis in the non-model fish *S. aurata*, genes were mapped to *Danio rerio* orthologs using Ensembl BioMart; when multiple orthologs existed, the best (% identity) match was retained. Over-representation analysis (ORA) was performed with *clusterProfiler* (GO Biological Process/Molecular Function/Cellular Component via enrichGO). The gene universe was the set of the expressed genes effectively mapped to zebrafish. Multiple testing was controlled with Benjamini–Hochberg (FDR). When no ORA terms passed strict FDR (FDR < 0.05), we ran pre-ranked GSEA using a signed statistic (sign(L2F) × − log10p). GSEA was performed with *clusterProfiler* (gseGO for GO BP/MF/CC; gseKEGG for “dre”), with default permutation settings, minimum gene-set size ≥ 10 (KEGG)and 15 (GO), and Benjamini–Hochberg FDR control. Redundant significant GO terms were reduced with *simplify* (semantic similarity cutoff 0.5) before visualization.

Additionally, a gene co-expression analysis (WGCNA [95]) was performed. Prior to network construction, sample clustering was evaluated and one outlier sample (Ctrol5) was excluded to preserve scale-free topology. Variance-stabilized/log-normalized expression matrices of the remaining samples were filtered to exclude genes with mean expression under 0.5, zero variance or missing values within experimental groups. The resulting set was further filtered to retain the top 10,000 most variable genes to ensure a robust, high-signal co-expression network across Control (*n* = 4) and Gen + VitC (*n* = 5) groups. Soft-thresholding power was chosen with *pickSoftThreshold*; signed adjacency (Additional file 2: Fig. S1) and signed TOM were used for network construction. To mitigate the risk of stochastic associations inherent in smaller datasets, we employed bi-weight midcorrelation (*bicor*) with an outlier constraint (maxPOutliers = 0.1). Modules were identified with *blockwiseModules* (using custom settings: deepSplit = 2, minModuleSize = 50, mergeCutHeight = 0.45). Module eigengenes (MEs) were computed with *moduleEigengenes* and correlated (Pearson) with diet and sample physiological traits, including liver weight, triglycerides, cholesterol, mitochondrial ROS, catalase, protein carbonylation, antioxidant capacity, plasma proteins, glucose, lactate, triglycerides, cholesterol, and cortisol; *p*-values were corrected with FDR. For functional interpretation of the WGCNA modules, the variation in biological pathway activity across individual samples was assessed through a Gene Set Variation Analysis (GSVA) using the Single-Sample Gene Set Enrichment Analysis (ssGSEA) method within the GSVA R package. Normalized gene expression counts were transformed into sample-wise enrichment scores for 1,468 gene sets derived from the Gene Ontology (GO) Biological Process and KEGG pathway enrichments. To ensure biological robustness, gene sets were restricted to those containing between 30 and 800 genes, and only pathways with a minimum of 20 genes represented in the experimental matrix were retained for enrichment scoring, including z-score standardization to visualize relative pathway activity across the Control (*n* = 4) and Gen + VitC (*n* = 5) groups. Pathways were filtered based on a global GSEA significance threshold of *q* < 0.05 and an absolute Normalized Enrichment Score (NES) ≥ 1.8. Finally, module-specific pathway activity was determined by calculating the Pearson correlation between sample-wise ssGSEA scores and the respective WGCNA module eigengenes. This approach allowed for the direct integration of pathway-level dynamics with our WGCNA framework by assessing the correlation between individual pathway enrichment scores and the respective module eigengenes.

### RRBS library preparation and sequencing

Genomic DNA (200 ng) was digested with MspI (NEB) and ligated to pre-annealed adapters containing 5-methyl-dC. Adapter-ligated fragments ≥ 50 bp were purified (DNA Clean & Concentrator-5), bisulfite converted (EZ DNA Methylation-Lightning), PCR-amplified, and purified prior to sequencing on an Illumina NovaSeq 6000 (150 bp paired-end).

### RRBS data processing and CpG methylation calling

Raw FASTQ files were trimmed with Trim Galore! using the RRBS mode (–rrbs) to remove adapters, filled-in bases at MspI sites, and low-quality tails. Read quality was assessed with FastQC. Reads were aligned to the *S. aurata* reference genome (fSpaAur1.1; GCF_900880675.1) using Bismark (Krueger and Andrews 2011) with Bowtie2 (Langmead and Salzberg 2012). PCR duplicates were removed, and per-CpG methylation was extracted with MethylDackel (https://github.com/dpryan79/MethylDackel). Per-sample CpG calls were read with methylKit [96] and quality-filtered (coverage ≥ 10; upper 0.1% coverage trimmed with filterByCoverage, then normalizeCoverage). CpG tables were united across samples with unite, and per-CpG β values (0–1) were computed from the united object (percMethylation/100). For gene- and promoter-level summaries used in some figures, CpGs were aggregated with regionCounts, followed by uniting and converting to β.

### Differential methylation analysis and region calling

To call site-level differentially methylated cytosines (DMCs) and regional differential methylation (DMRs), we used DSS [97], which models bisulfite counts with a beta–binomial regression and applies local smoothing/dispersion shrinkage. The filtered and normalized methylKit object was converted to a bsseq object (per-site methylated counts and coverages, all samples aligned to the union of CpG coordinates). DSS was run on this bsseq object (*DMLtest*, smoothing with a default span of 500 bp), and DMCs were called with *callDML* (thresholds: |Δβ|≥ 0.20 and nominal P ≤ 0.05), then filtered at FDR < 0.05. DMRs were called with *callDMR* (nominal P ≤ 0.05, |Δβ|≥ 0.20, minlen = 50 and dis.merge = 50), returning regions with posterior support for the specified effect size. All DMCs/DMRs were converted to *GRanges* and functionally annotated by overlap (promoter, gene body or intergenic). Unless stated otherwise, CpG-level results were summarized at gene and promoter scales for integrative analyses.

### Genomic feature definitions

Gene models were imported from the species GFF3 (*rtracklayer*). Promoters were defined with *promoters (upstream* = *2000, downstream* = *200)*, resulting in regions − 2 kb to + 200 bp around the TSS without overlapping neighboring genes. Gene bodies were defined starting at the gene coordinates with the TSS-proximal 200 bp trimmed off (for “ + ” strands start = TSS + 200; for “ − ” strands end = TSS − 200); features < 200 bp were discarded. CpG methylation calls (β converted to M-values with an ε = 1e − 6) were overlapped to promoter and gene-body ranges (*GenomicRanges*). For each feature in each sample, values were summarized as the column median across overlapping CpGs (single-CpG features used that value). Optional annotations such as lncRNAs, transposable elements, UTRs, and CpG islands were obtained from existing datasets for the species as in [75].

### Functional enrichment of differentially methylated genes

Gene sets (4) with overlapping DMCs (> 5) or DMRs within promoter or extended gene regions (gene body + −2 kb to + 200 bp promoter), were analyzed with over-representation analysis (ORA) using clusterProfiler to identify biological themes in Gene Ontology (GO) and KEGG pathways relative to a background set of all probed genes. To ensure physiological relevance to the study tissue, significant terms (q < 0.1 and minGSSize > 10) were further refined using a custom filtering strategy to prioritize liver-specific processes while excluding irrelevant neurological terms. Semantic simplification (described before) was used to reduce GO term redundancy.

### DNA methylation–gene expression integration

CpG-level DMCs (Δβ in percentage points; 100 × DSS “diff” when needed) were overlapped to “expanded genes” (gene body + promoter) and promoter regions. For gene-wise summaries (DMGs) we required ≥ 5 overlapping DMCs per gene region, computed sample-level mean β across overlapping CpGs, and derived per-gene Δβ (Gen + VitC − Control). Scatterplots contrasted per-CpG Δβ against gene L2F for (i) DEGs and (ii) all expressed genes (classification into Hypo/Hyper × Up/Down used |Δβ|≥ 15 and | L2F |≥ 1). Global coupling was also assessed by binning genes by expression and regressing mean β vs expression per group. Heatmaps display per-gene β (samples as columns; Control vs Gen + VitC) for DMC-enriched promoters and gene bodies after filtering by minimum DMC count and minimum per-gene Δβ (typically ≥ 0.20 in β units) with Wilcoxon P ≤ 0.05.

We finally inferred epigenetic–transcriptional modules with a FEM-style workflow adapted for RRBS and non-model species and used before in *S. aurata* [75, 98, 99]. Briefly, we built a gilthead seabream protein–protein interaction (PPI) graph from STRING v12 (species 8175; accessed Jun 2025), reduced to 2,673 nodes and 34,502 high-confidence edges (combined_score ≥ 95th percentile). By utilizing a robust interactome rather than a sparse one, we reduce the risk of detecting unstable or stochastic modules, ensuring that the reported epigenetic hotspots represent consistent biological signatures. STRING protein IDs were mapped to Ensembl gene IDs via Ensembl BioMart (release 114), duplicates collapsed, and isolates removed to yield a gene-level adjacency matrix. Differentially methylated CpGs from DSS were overlapped with gene bodies expanded ± 2 kb and with −2 kb to + 200 bp promoters to derive two DMC-supported gene sets. Using these as inputs, we applied our RRBS FEM routine (*RunRRBSFEM*) to score subnetworks around candidate hubs by integrating per-gene/promoter methylation contrasts (β: Gen + VitC vs Control) with RNA-seq differential expression, and we summarized top-ranked modules for downstream inspection.

### Air exposure challenge and mitochondrial reactive oxygen species in liver

Fish were fasted for 24 h. Afterwards, 12 fish were immediately sampled (non-stressed) while 7 fish were air- exposed for 3 min, placed in aerated tanks with clean seawater and sampled (stressed) after 1 h of recovery. Then, fish were sacrificed with an anesthetic overdose (1 mL L^−1^ of 2-phenoxyethanol) and liver samples were collected and stored at −80 ◦C. For mitochondrial reactive oxygen species (mtROS) determination, samples were homogenized in ice-cold mitochondria isolation buffer (225 mM mannitol, 75 mM sucrose, 1 mM EGTA and 4 mM HEPES, pH 7.2). Then, the homogenate was centrifuged for 10 min at 600 g and 4 ◦C. The supernatant was centrifuged for 10 min, at 11,000 g and 4 ◦C. The pellet was resuspended in a buffer containing 250 mM sucrose and 5 mM HEPES (pH 7.2). mtROS production was assessed by the dihydrodichloro-fluorescein diacetate method, H(2)DCF- at 529 nm (emission) and 503 nm (excitation) as in our previous work [35]. Results were expressed as Relative Fluorescence Units (RFU) per mg mitochondrial protein. The protein content was determined by the Bradford method using BSA as the standard. Determinations were made in 96 well flat bottom microplates using a microplate reader (Synergy H1, BioTek Instrument, Inc., USA). Outliers were identified using the ROUT (Robust Regression and Outlier Removal) method implemented in GraphPad Prism, with the false discovery rate parameter set at Q = 1%. This conservative threshold minimizes the probability of incorrectly classifying valid observations as outliers, while only remove extreme observations.

## Supplementary Information

Supplementary Material 1: Additional file 1. Supplementary Table ST1. RNA-seq sequencing and mapping statistics for each sample. Supplementary Table ST2. Differential gene expression results from DESeq2 with normalized read counts per sample. Supplementary Table ST3. Gene ontology functional enrichment analysis of up- and down-regulated genes. Supplementary Table ST4. RRBS quality metrics and CpG coverage statistics per sample. Supplementary Table ST5. Differentially methylated regions (DMRs) identified by DSS. Supplementary Table ST6. Functional enrichment analysis of differential methylation. Supplementary Table ST7. Functional Epigenetic Module (FEM) analysis integrating DNA methylation and gene expression across gene networks

Supplementary Material 2: Additional file 2: Figure S1: WGCNA network topology diagnostics. Analysis of soft-thresholding power selection for signed network construction using biweight midcorrelation. (A) Scale-free fit index (R^2) as a function of the soft-thresholding power; a power of 13 (black) was selected to achieve a scale-free topology fit R^2 > 0.85. (B) Mean connectivity as a function of the soft-thresholding power. Figure S2: Integration of pathway-level activity with WGCNA module eigengenes. Dotplot illustrating the correlation between sample-wise enrichment scores (ES) and module eigengenes (ME) for the Red, Turquoise, and Blue modules. Pathway scores were calculated using the Single Sample Gene Set Enrichment Analysis (ssGSEA) method within the GSVA R package, utilizing Gene Ontology (GO) gene sets mapped to zebrafish orthologs. Displayed terms were restricted to those achieving a global GSEA significance of q < 0.05 and an absolute Normalized Enrichment Score (NES) > 1.8, with arrows indicating the direction of treatment effect (Up/Down). Point size represents the absolute Pearson correlation coefficient (|r|) between the ES and ME, while color indicates the statistical significance (-log10 FDR) of the association. Figure S3. General characterization of DNA methylation patterns in gilthead seabream liver. DNA methylation levels (average beta) in and around TSS and DNA methylation levels (average beta) in the context of CpG islands across the genome. Below: Relationship between DNA methylation and gene expression across genomic regions: Gene Body + Promoter (-2 kb to +200 bp from TSS), Promoter only, Gene body only. Figure S4: Frequency of Differentially methylated regions (DMR) overlapping genomic regions. Figure S5: Heatmap of DNA methylation levels (ß) per sample for DMC enriched promoters (only promoters with > 5 DMCs). Figure S6: Heatmap of DNA methylation levels (ß) per sample for DMC enriched extended genes (gene body + promoter with > 5 DMCs). Genes highlighted in red were also differentially expressed (DEGs).

## Data Availability

The datasets generated and analyzed during the current study are available for download from NCBI with BioProject ID PRJNA1425545. Datasets will be made public after acceptance.

## References

[1] Chowdhury S, Saikia SK. Oxidative stress in fish: a review. J Sci Res. 2020;12(1):145–60.

[2] Olsvik PA, Torstensen BE, Hemre GI, Sanden M, Waagbø R. Hepatic oxidative stress in Atlantic salmon (Salmo salar L.) transferred from a diet based on marine feed ingredients to a diet based on plant ingredients. Aquac Nutr. 2011;17:e424–36. 10.1111/j.1365-2095.2010.00778.x.

[3] He L, Zhang Y, Cao Q, Shan H, Zong J, Feng L, et al. Hepatic oxidative stress and cell death influenced by dietary lipid levels in a fresh teleost. Antioxidants (Basel). 2024;13(7):808. 10.3390/antiox13070808. 39061877 PMC11273915

[4] Ibarz A, Martín-Pérez M, Blasco J, Bellido D, de Oliveira E, Fernández-Borràs J. Gilthead sea bream liver proteome altered at low temperatures by oxidative stress. Proteomics. 2010;10(5):963–75. 10.1002/pmic.200900528. 20131326

[5] Wang Y, Li C, Pan C, Liu E, Zhao X, Ling Q. Alterations to transcriptomic profile, histopathology, and oxidative stress in liver of pikeperch (Sander lucioperca) under heat stress. Fish Shellfish Immunol. 2019;95:659–69. 10.1016/j.fsi.2019.11.014. 31706008

[6] Dettleff P, Zuloaga R, Fuentes M, Gonzalez P, Aedo J, Estrada JM, et al. High-temperature stress effect on the red cusk-eel (Geypterus chilensis) liver: transcriptional modulation and oxidative stress damage. Biology. 2022;11(7):990. 10.3390/biology11070990. 36101373 PMC9312335

[7] Guo Y, Jin C, Wei C, Zhong K, Gao Y, Li P, et al. The effects of acute temperature changes on transcriptomic responses in the liver of leopard coral groupers (Plectropomus leopardus). Antioxidants (Basel). 2025;14(2):223. 10.3390/antiox14020223. 40002409 PMC11851849

[8] Messina CM, Manuguerra S, Arena R, Espinosa-Ruiz C, Curcuraci E, Esteban MA, et al. Contaminant-induced oxidative stress underlies biochemical, molecular and fatty acid profile changes, in gilthead seabream (Sparus aurata L.). Res Vet Sci. 2023;159:244–51. 10.1016/j.rvsc.2023.04.021. 37178628

[9] Ghannam HE, Khedr AI, El-Sayed R, et al. Oxidative stress responses and histological changes in the liver of Nile tilapia exposed to silver bulk and nanoparticles. Sci Rep. 2025;15:15390. 10.1038/s41598-025-97731-8. 40316619 PMC12048537

[10] Duan Y, Xiao M, Zhu R, Nan Y, Yang Y, Huang X, et al. Effects of ammonia stress on the antioxidant, ferroptosis, and immune response in the liver of golden pompano Trachinotus ovatus. Antioxidants (Basel). 2025;14(4):419. 10.3390/antiox14040419. 40298705 PMC12024250

[11] Teles M, Oliveira M, Jerez-Cepa I, Franco-Martínez L, Tvarijonaviciute A, Tort L, et al. Transport and recovery of gilthead sea bream (Sparus aurata L.) sedated with clove oil and MS222: effects on oxidative stress status. Front Physiol. 2019;10:523. 10.3389/fphys.2019.00523. 31130870 PMC6509202

[12] Martos-Sitcha JA, Mancera JM, Calduch-Giner JA, Yúfera M, Martínez-Rodríguez G, Pérez-Sánchez J. Unraveling the tissue-specific gene signatures of gilthead sea bream (Sparus aurata L.) after hyper- and hypo-osmotic challenges. PLoS ONE. 2016;11(2):e0148113. 10.1371/journal.pone.0148113. 26828928 PMC4734831

[13] Pascual P, Pedrajas JR, Toribio F, López-Barea J, Peinado J. Effect of food deprivation on oxidative stress biomarkers in fish (Sparus aurata). Chem Biol Interact. 2003;145:191–9. 12686495 10.1016/s0009-2797(03)00002-4

[14] Birnie-Gauvin K, Costantini D, Cooke SJ, Willmore WG. A comparative and evolutionary approach to oxidative stress in fish: A review. Fish Fish. 2017;18:928–42. 10.1111/faf.12215.

[15] Menon SV, Kumar A, Middha SK, Paital B, Mathur S, Johnson R, et al. Water physicochemical factors and oxidative stress physiology in fish, a review. Front Environ Sci. 2023;11:1240813. 10.3389/fenvs.2023.1240813.

[16] Rychen G, Aquilina G, Azimonti G, Bampidis V, Bastos ML, Bories G, et al. Safety and efficacy of butylated hydroxyanisole (BHA) as a feed additive for all animal species. EFSA J. 2018;16:e05215. 10.2903/j.efsa.2018.5215. 32625845 PMC7009650

[17] Hoseinifar SH, Yousefi S, Van Doan H, Ashouri G, Gioacchini G, Maradonna F, et al. Oxidative stress and antioxidant defense in fish: the implications of probiotic, prebiotic, and synbiotics. Rev Fish Sci Aquacult. 2020;29(2):198–217. 10.1080/23308249.2020.1795616.

[18] Reis B, Gonçalves AT, Santos P, Sardinha M, Conceição LEC, Serradeiro R, et al. Immune status and hepatic antioxidant capacity of gilthead seabream Sparus aurata juveniles fed yeast and microalga derived β-glucans. Mar Drugs. 2021;19(12):653. 10.3390/md19120653. 34940652 PMC8704051

[19] Hu X, Ma W, Zhang D, Tian Z, Yang Y, Huang Y, et al. Application of natural antioxidants as feed additives in aquaculture: a review. Biology. 2025;14(1):87. 10.3390/biology14010087. 39857317 PMC11762552

[20] Pereira A, Marmelo I, Chainho T, Bolotas D, Dias M, Cereja R, et al. Improving farmed juvenile gilthead seabream (Sparus aurata) stress response to marine heatwaves and vibriosis through seaweed-based dietary modulation. Animals (Basel). 2025;15(13):1970. 10.3390/ani15131970. 40646869 PMC12248664

[21] Hunyadi A. The mechanism(s) of action of antioxidants: From scavenging reactive oxygen/nitrogen species to redox signaling and the generation of bioactive secondary metabolites. Med Res Rev. 2019;39(6):2505–33. 10.1002/med.21592. 31074028

[22] Kreuz S, Fischle W. Oxidative stress signaling to chromatin in health and disease. Epigenomics. 2016;8(6):843–62. 10.2217/epi-2016-0002. 27319358 PMC5619053

[23] Niu Y, DesMarais TL, Tong Z, Yao Y, Costa M. Oxidative stress alters global histone modification and DNA methylation. Free Radic Biol Med. 2015;82:22–8. 10.1016/j.freeradbiomed.2015.01.028. 25656994 PMC4464695

[24] Seddon AR, Das AB, Hampton MB, Stevens AJ. Site-specific decreases in DNA methylation in replicating cells following exposure to oxidative stress. Hum Mol Genet. 2023;32(4):632–48. 10.1093/hmg/ddac232. 36106794 PMC9896486

[25] Le X, Mu J, Peng W, Tang J, Xiang Q, Tian S, et al. DNA methylation downregulated ZDHHC1 suppresses tumor growth by altering cellular metabolism and inducing oxidative/ER stress-mediated apoptosis and pyroptosis. Theranostics. 2020;10(21):9495–511. 10.7150/thno.45631. 32863941 PMC7449911

[26] Wang XC, Zhang YS, Ling H, You JB, Cheng J, Liu ZY, et al. Epigenetic silencing of SOD2 exacerbates mitochondrial oxidative stress and promotes pulmonary fibrosis. Free Radic Biol Med. 2025;235:176–89. 10.1016/j.freeradbiomed.2025.04.034. 40280315

[27] Guo Y, Yu S, Zhang C, Kong AN. Epigenetic regulation of Keap1-Nrf2 signaling. Free Radic Biol Med. 2015;88(Pt B):337–49. 10.1016/j.freeradbiomed.2015.06.013. 26117320 PMC4955581

[28] Gornicka A, Morris-Stiff G, Thapaliya S, Papouchado BG, Berk M, Feldstein AE. Transcriptional profile of genes involved in oxidative stress and antioxidant defense in a dietary murine model of steatohepatitis. Antioxid Redox Signal. 2011;15(2):437–45. 10.1089/ars.2010.3815. 21194384 PMC3118609

[29] Adam AC, Saito T, Espe M, Whatmore P, Fernandes JMO, Vikeså V, et al. Metabolic and molecular signatures of improved growth in Atlantic salmon (Salmo salar) fed surplus levels of methionine, folic acid, vitamin B6 and B12 throughout smoltification. Br J Nutr. 2022;127(9):1289–302. 10.1017/S0007114521002336. 34176547

[30] Robea MA, Ilie OD, Nicoara MN, Solcan G, Romila LE, Ureche D, et al. Vitamin B12 ameliorates pesticide-induced sociability impairment in zebrafish (Danio rerio): a prospective controlled intervention study. Animals. 2024;14(3):405. 10.3390/ani14030405. 38338046 PMC10854844

[31] Skjærven KH, Adam AC, Saito T, Waagbø R, Espe M. Epigenetics in Fish Nutritional Programming, in Epigenetics in Aquaculture, eds F. Piferrer and H.-P. Wang (2023), 231–244, 10.1002/9781119821946.ch10

[32] Seisenberger S, Peat JR, Hore TA, Santos F, Dean W, Reik W. Reprogramming DNA methylation in the mammalian life cycle: building and breaking epigenetic barriers. Philos Trans R Soc Lond B Biol Sci. 2013;368(1609):20110330. 10.1098/rstb.2011.0330. 23166394 PMC3539359

[33] Win W, Cao Z, Peng X, Trush MA, Li Y. Different effects of genistein and resveratrol on oxidative DNA damage in vitro. Mutat Res. 2002;513(1–2):113–20. 10.1016/s1383-5718(01)00303-5. 11719096

[34] Zhang K, Wang J, Xu B. Critical review on molecular mechanisms for genistein’s beneficial effects on health through oxidative stress reduction. Antioxidants (Basel). 2025;14(8):904. 10.3390/antiox14080904. 40867803 PMC12382804

[35] Navarro-Guillén C, Huesa-Cerdán R, Hidalgo-Pérez JA, Simó-Mirabet P, Rodríguez-Viera L, Martos-Sitcha JA, et al. One-carbon nutrients and genistein as nutritional programming effectors in juvenile gilthead seabream (Sparus aurata): Contrasting effects on phenotypic traits. Aquaculture. 2025;598:742063.

[36] Oseni SO, Kumi-Diaka J, Branly R, et al. Pyroelectrically generated very low dose ionizing radiation enhances chemopreventive and chemotherapeutic effects of genistein isoflavone in human prostate cancer cells. J Cancer Prev Curr Res. 2014;1:1–14. 10.15406/jcpcr.2014.01.00010.

[37] Fritz H, Flower G, Weeks L, et al. Intravenous vitamin C and cancer: a systematic review. Integr Cancer Ther. 2014;13:280–300. 10.1177/1534735414534463. 24867961

[38] Xie Q, Bai Q, Zou LY, Zhang QY, Zhou Y, Chang H, et al. Genistein inhibits DNA methylation and increases expression of tumor suppressor genes in human breast cancer cells. Genes Chromosom Cancer. 2014;53:422–31. 10.1002/gcc.22154. 24532317

[39] Ionescu VS, Popa A, Alexandru A, Manole E, Neagu M, Pop S. Dietary phytoestrogens and their metabolites as epigenetic modulators with impact on human health. Antioxidants (Basel). 2021;10:1893. 10.3390/antiox10121893. 34942997 PMC8750933

[40] Sundaram MK, Unni S, Somvanshi P, Bhardwaj T, Mandal RK, Hussain A, et al. Genistein modulates signaling pathways and targets several epigenetic markers in HeLa cells. Genes. 2019;10:955. 10.3390/genes10120955. 31766427 PMC6947182

[41] Gerecke C, Schumacher F, Edlich A, Wetzel A, Yealland G, Neubert LK, et al. Vitamin C promotes decitabine or azacytidine induced DNA hydroxymethylation and subsequent reactivation of the epigenetically silenced tumour suppressor CDKN1A in colon cancer cells. Oncotarget. 2018;9:32822–40. 10.18632/oncotarget.25999. 30214687 PMC6132357

[42] Pavlovic V, Ciric M, Petkovic M, Golubovic M. Vitamin C and epigenetics: A short physiological overview. Open Med (Wars). 2023;18:20230688. 10.1515/med-2023-0688. 37359134 PMC10290282

[43] Wang L, Li A, Liu Y, Zhan S, Zhong L, Du Y, et al. Genistein protects against acetaminophen-induced liver toxicity through augmentation of SIRT1 with induction of Nrf2 signalling. Biochem Biophys Res Commun. 2020;527(1):90–7. 10.1016/j.bbrc.2020.04.100. 32446397

[44] Peng D, Ge G, Gong Y, Zhan Y, He S, Guan B, et al. Vitamin C increases 5-hydroxymethylcytosine level and inhibits the growth of bladder cancer. Clin Epigenet. 2018;10(1):94–107. 10.1186/s13148-018-0527-7. PMC6045833 30005692

[45] Stincone A, Prigione A, Cramer T, Wamelink MM, Campbell K, Cheung E, et al. The return of metabolism: biochemistry and physiology of the pentose phosphate pathway. Biol Rev Camb Philos Soc. 2015;90(3):927–63. 10.1111/brv.12140. 25243985 PMC4470864

[46] Ducker GS, Rabinowitz JD. One-carbon metabolism in health and disease. Cell Metab. 2017;25(1):27–42. 10.1016/j.cmet.2016.08.009. 27641100 PMC5353360

[47] Ryter SW, Bhatia D, Choi ME. Autophagy: a lysosome-dependent process with implications in cellular redox homeostasis and human disease. Antioxid Redox Signal. 2019;30(1):138–59. 10.1089/ars.2018.7518. 29463101 PMC6251060

[48] Dubois V, Gheeraert C, Vankrunkelsven W, Dubois-Chevalier J, Dehondt H, Bobowski-Gerard M, et al. Endoplasmic reticulum stress actively suppresses hepatic molecular identity in damaged liver. Mol Syst Biol. 2020;16(5):e9156. 10.15252/msb.20199156. 32407006 PMC7224309

[49] Huang Z, Ma A, Yang S, Liu X, Zhao T, Zhang J, et al. Transcriptome analysis and weighted gene co-expression network reveals potential genes responses to heat stress in turbot Scophthalmus maximus. Comp Biochem Physiol Part D Genomics Proteomics. 2020;33:100632. 10.1016/j.cbd.2019.100632. 31715507

[50] Xing M, Rong Z, Zhao X, Gao X, Hou Z, Zhang L, et al. Transcriptome analysis reveals hypoxic response key genes and modules as well as adaptive mechanism of crucian carp (Carassius auratus) gill under hypoxic stress. Front Immunol. 2025;5(16):1543605. 10.3389/fimmu.2025.1543605. PMC11835930 39975546

[51] Sobajima T, Yoshimura SI, Maeda T, Miyata H, Miyoshi E, Harada A. The Rab11-binding protein RELCH/KIAA1468 controls intracellular cholesterol distribution. J Cell Biol. 2018;217(5):1777–96. 10.1083/jcb.201709123. 29514919 PMC5940305

[52] Hernández-Oliveras A, Izquierdo-Torres E, Hernández-Martínez G, Zarain-Herzberg Á, Santiago-García J. Transcriptional and epigenetic landscape of Ca2+-signaling genes in hepatocellular carcinoma. J Cell Commun Signal. 2021;15(3):433–45. 10.1007/s12079-020-00597-w. 33398721 PMC8222487

[53] Kotzbauer PT, Lampe PA, Heuckeroth RO, Golden JP, Creedon DJ, Johnson EM Jr, et al. Neurturin, a relative of glial-cell-line-derived neurotrophic factor. Nature. 1996;384(6608):467–70. 10.1038/384467a0. 8945474

[54] Aizawa S, Fujiwara Y, Contu VR, Hase K, Takahashi M, Kikuchi H, et al. Lysosomal putative RNA transporter SIDT2 mediates direct uptake of RNA by lysosomes. Autophagy. 2016;12(3):565–78. 10.1080/15548627.2016.1145325. 27046251 PMC4836006

[55] Di Nunzio G, Hellberg S, Zhang Y, Ahmed O, Wang J, Zhang X, et al. Kupffer cells dictate hepatic responses to the atherogenic dyslipidemic insult. Nat Cardiovasc Res. 2024;3(3):356–71. 10.1038/s44161-024-00448-6. 39196121 PMC11358021

[56] Lee Y, Nguyen TL, Roh H, Kim A, Park J, Lee JY, et al. Mechanisms underlying probiotic effects on neurotransmission and stress resilience in fish via transcriptomic profiling. Fish Shellfish Immunol. 2023;141:109063. 10.1016/j.fsi.2023.109063. 37678478

[57] Rossi J, Herzig KH, Võikar V, Hiltunen PH, Segerstråle M, Airaksinen MS. Alimentary tract innervation deficits and dysfunction in mice lacking GDNF family receptor alpha2. J Clin Invest. 2003;112(5):707–16. 10.1172/JCI17995. 12952919 PMC182204

[58] Higgins MG, Fitzsimons C, McClure MC, McKenna C, Conroy S, Kenny DA, et al. GWAS and eQTL analysis identifies a SNP associated with both residual feed intake and GFRA2 expression in beef cattle. Sci Rep. 2018;8(1):14301. 10.1038/s41598-018-32374-6. 30250203 PMC6155370

[59] van Wijnen AJ, Bagheri L, Badreldin AA, Larson AN, Dudakovic A, Thaler R, et al. Biological functions of chromobox (CBX) proteins in stem cell self-renewal, lineage-commitment, cancer and development. Bone. 2021;143:115659. 10.1016/j.bone.2020.115659. 32979540

[60] Chang Y, Sun L, Kokura K, Horton JR, Fukuda M, Espejo A, et al. MPP8 mediates the interactions between DNA methyltransferase Dnmt3a and H3K9 methyltransferase GLP/G9a. Nat Commun. 2011;2:533. 10.1038/ncomms1549. 22086334 PMC3286832

[61] Jacquet K, Fradet-Turcotte A, Avvakumov N, Lambert JP, Roques C, Pandita RK, et al. The TIP60 complex regulates bivalent chromatin recognition by 53BP1 through direct H4K20me binding and H2AK15 acetylation. Mol Cell. 2016;62(3):409–21. 10.1016/j.molcel.2016.03.031. 27153538 PMC4887106

[62] Wu G, Fang YZ, Yang S, Lupton JR, Turner ND. Glutathione metabolism and its implications for health. J Nutr. 2004;134(3):489–92. 10.1093/jn/134.3.489. 14988435

[63] Vancura P, Csicsely E, Leiser A, Iuvone PM, Spessert R. Rhythmic regulation of photoreceptor and RPE genes important for vision and genetically associated with severe retinal diseases. Invest Ophthalmol Vis Sci. 2018;59(10):3789–99. 10.1167/iovs.18-24558. 30073352 PMC6071477

[64] Zhang M, Li Q, Yang T, Meng F, Lai X, Liang L, et al. Positive feedback between retinoic acid and 2-phospho-L-ascorbic acid trisodium salt during somatic cell reprogramming. Cell Regen. 2020;9(1):17. 10.1186/s13619-020-00057-1. 33000315 PMC7527398

[65] Ghyselinck NB, Duester G. Retinoic acid signaling pathways. Development. 2019;146(13):dev167502. 10.1242/dev.167502. PMC6633611 31273085

[66] Klyuyeva AV, Belyaeva OV, Goggans KR, Krezel W, Popov KM, Kedishvili NY. Changes in retinoid metabolism and signaling associated with metabolic remodeling during fasting and in type I diabetes. J Biol Chem. 2021;296:100323. 10.1016/j.jbc.2021.100323. 33485967 PMC7949101

[67] Skjærven KH, Jakt LM, Fernandes JMO, Dahl JA, Adam AC, Klughammer J, et al. Parental micronutrient deficiency distorts liver DNA methylation and expression of lipid genes associated with a fatty-liver-like phenotype in offspring. Sci Rep. 2018;8:3055. 10.1038/s41598-018-21211-5. 29445184 PMC5812986

[68] Maag D, Maxwell MJ, Hardesty DA, Boucher KL, Choudhari N, Hanno AG, et al. Inositol polyphosphate multikinase is a physiologic PI3-kinase that activates Akt/PKB. Proc Natl Acad Sci U S A. 2011;108(4):1391–6. 10.1073/pnas.1017831108. 21220345 PMC3029688

[69] Dejeans N, Tajeddine N, Beck R, Verrax J, Taper H, Gailly P, et al. Endoplasmic reticulum calcium release potentiates the ER stress and cell death caused by an oxidative stress in MCF-7 cells. Biochem Pharmacol. 2010;79(9):1221–30. 10.1016/j.bcp.2009.12.009. 20006589

[70] Andrejeva G, Gowan S, Lin G, Wong Te Fong AL, Shamsaei E, Parkes HG, et al. *De novo* phosphatidylcholine synthesis is required for autophagosome membrane formation and maintenance during autophagy. Autophagy. 2020;16(6):1044–60. 10.1080/15548627.2019.1659608. 31517566 PMC7469489

[71] Liu Y, Wu Y, Jiang M. The emerging roles of PHOSPHO1 and its regulated phospholipid homeostasis in metabolic disorders. Front Physiol. 2022;13:935195. 10.3389/fphys.2022.935195. 35957983 PMC9360546

[72] Horibata Y, Hirabayashi Y. Identification and characterization of human ethanolaminephosphotransferase1. J Lipid Res. 2007;48(3):503–8. 10.1194/jlr.C600019-JLR200. 17132865

[73] Fritz KS, Petersen DR. Exploring the biology of lipid peroxidation-derived protein carbonylation. Chem Res Toxicol. 2011;24(9):1411–9. 10.1021/tx200169n. 21812433 PMC3178011

[74] Perera E, Román-Padilla J, Hidalgo-Pérez JA, Huesa-Cerdán R, Yúfera M, Mancera JM, et al. Tissue explants as tools for studying the epigenetic modulation of the GH-IGF-I axis in farmed fish. Front Physiol. 2024;15:1410660. 10.3389/fphys.2024.1410660. 38966230 PMC11222784

[75] Perera E, Navarro-Guillén C, Román-Padilla J, Huesa-Cerdán R, Hidalgo-Perez JA, Martos-Sitcha JA, et al. Decitabine-induced DNA methylation remodeling reveals targetable biological processes in gilthead seabream pituitary and liver explants. Epigenetics. 2025;20:2566515. 10.1080/15592294.2025.2566515. 41056485 PMC12505512

[76] Gidlöf O, Johnstone AL, Bader K, Khomtchouk BB, O’Reilly JJ, Celik S, et al. Ischemic preconditioning confers epigenetic repression of Mtor and induction of autophagy through G9a-dependent H3K9 dimethylation. J Am Heart Assoc. 2016;5(12):e004076. 10.1161/JAHA.116.004076. 28007739 PMC5210409

[77] Owings KG, Chow CY. A Drosophila screen identifies a role for histone methylation in ER stress preconditioning. G3 (Bethesda). 2024;14(2):jkad265. 10.1093/g3journal/jkad265. 38098286 PMC11021027

[78] Chen Y, Dong H, Thompson DC, Shertzer HG, Nebert DW, Vasiliou V. Glutathione defense mechanism in liver injury: insights from animal models. Food Chem Toxicol. 2013;60:38–44. 10.1016/j.fct.2013.07.008. 23856494 PMC3801188

[79] Hong SH, Yu X, Zhu Y, Chen Y. Liver epigenomic signature associated with chronic oxidative stress in a mouse model of glutathione deficiency. Chem Biol Interact. 2024;398:111093. 10.1016/j.cbi.2024.111093. 38830566 PMC11223951

[80] Saito T, Whatmore P, Taylor JF, et al. Micronutrient supplementation affects DNA methylation in male gonads with potential intergenerational epigenetic inheritance involving the embryonic development through glutamate receptor-associated genes. BMC Genomics. 2022;23(1):115. 10.1186/s12864-022-08348-4. 35144563 PMC8832813

[81] Saito T, Espe M, Vikeså V, et al. One-carbon metabolism nutrients impact the interplay between DNA methylation and gene expression in liver, enhancing protein synthesis in Atlantic salmon. Epigenetics. 2024;19(1):2318517. 10.1080/15592294.2024.2318517. 38404006 PMC10900267

[82] Skjærven et al., 2022. Chapter 5 - Nutritional epigenetics. Cellular and Molecular Approaches in Fish Biology. Elsevier Inc. 10.1016/B978-0-12-822273-7.00006-9

[83] Dhanasiri AKS, Siciliani D, Kortner TM, et al. Epigenetic changes in pyloric caeca of Atlantic salmon fed diets containing increasing levels of lipids and choline. Epigenetics. 2024;19(1):2305079. 10.1080/15592294.2024.2305079. 38281164 PMC10824149

[84] Calduch-Giner JA, Davey G, Saera-Vila A, et al. Use of microarray technology to assess the time course of liver stress response after confinement exposure in gilthead sea bream (Sparus aurata L.). BMC Genomics. 2010;11:193. 10.1186/1471-2164-11-193. 20307314 PMC2860363

[85] Krueger F. 2012. Trim Galore: A Wrapper Tool around Cutadapt and FastQC to Consistently Apply Quality and Adapter Trimming to FastQ Files, with Some Extra Functionality for MspI-Digested RRBS-Type (Reduced Representation Bisufite-Seq) Libraries. URL https://www.Bioinformatics.Babraham.Ac.Uk/Projects/Trim_galore/. (Date of Access: 28/04/2016).

[86] Dobin A, Davis CA, Schlesinger F, Drenkow J, Zaleski C, Jha S, et al. STAR: ultrafast universal RNA-seq aligner. Bioinformatics. 2013;29(1):15–21. 10.1093/bioinformatics/bts635. 23104886 PMC3530905

[87] Patel, Harshil, Jonathan Manning, Phil Ewels, Maxime U. Garcia, Alexander Peltzer, Rickard Hammarén, Olga Botvinnik, et al. 2025. Nf-Core/Rnaseq: Nf-Core/Rnaseq v3.19.0 - Tungsten Turtle. Zenodo. 10.5281/ZENODO.1400710.

[88] Danecek P, Bonfield JK, Liddle J, Marshall J, Ohan V, Pollard MO, et al. Twelve years of SAMtools and BCFtools. Gigascience. 2021;10(2):giab008. 10.1093/gigascience/giab008. 33590861 PMC7931819

[89] Wang L, Wang S, Li W. RSeQC: quality control of RNA-seq experiments. Bioinformatics. 2012;28(16):2184–5. 10.1093/bioinformatics/bts356. 22743226

[90] García-Alcalde F, Okonechnikov K, Carbonell J, Cruz LM, Götz S, Tarazona S, et al. Qualimap: evaluating next-generation sequencing alignment data. Bioinformatics. 2012;28(20):2678–9. 10.1093/bioinformatics/bts503. 22914218

[91] Daley T, Smith AD. Predicting the molecular complexity of sequencing libraries. Nat Methods. 2013;10(4):325–7. 10.1038/nmeth.2375. 23435259 PMC3612374

[92] Sayols S, Scherzinger D, Klein H. dupRadar: a Bioconductor package for the assessment of PCR artifacts in RNA-Seq data. BMC Bioinformatics. 2016;17:428. 10.1186/s12859-016-1276-2. 27769170 PMC5073875

[93] Liao Y, Smyth GK, Shi W. featureCounts: an efficient general purpose program for assigning sequence reads to genomic features. Bioinformatics. 2014;30(7):923–30. 10.1093/bioinformatics/btt656. 24227677

[94] Love MI, Huber W, Anders S. Moderated estimation of fold change and dispersion for RNA-seq data with DESeq2. Genome Biol. 2014;15(12):550. 10.1186/s13059-014-0550-8. 25516281 PMC4302049

[95] Langfelder P, Horvath S. WGCNA: an R package for weighted correlation network analysis. BMC Bioinformatics. 2008;9:559. 10.1186/1471-2105-9-559. 19114008 PMC2631488

[96] Akalin A, Kormaksson M, Li S, Garrett-Bakelman FE, Figueroa ME, Melnick A, et al. methylKit: a comprehensive R package for the analysis of genome-wide DNA methylation profiles. Genome Biol. 2012;13(10):R87. 10.1186/gb-2012-13-10-r87. 23034086 PMC3491415

[97] Feng H, Wu H. Differential methylation analysis for bisulfite sequencing using DSS. Quant Biol. 2019;7(4):327–34. 10.1007/s40484-019-0183-8. 34084562 PMC8171293

[98] Jiao Y, Widschwendter M, Teschendorff AE. A systems-level integrative framework for genome-wide DNA methylation and gene expression data identifies differential gene expression modules under epigenetic control. Bioinformatics. 2014;30(16):2360–6. 10.1093/bioinformatics/btu316. 24794928

[99] Owen N, Toms M, Tian Y, Toualbi L, Richardson R, Young R, et al. Loss of the crumbs cell polarity complex disrupts epigenetic transcriptional control and cell cycle progression in the developing retina. J Pathol. 2023;259(4):441–54. 10.1002/path.6056. 36656098 PMC10601974

