# Supplementary material for: Mild oxidative stress and dietary epigenetic modulators direct DNA methylation remodeling toward stress-resilience pathways
Source: BMC Genomics. 2026 Jul 3;27:779. doi: 10.1186/s12864-026-13088-w (PMC13599118; doi:10.1186/s12864-026-13088-w)
Supplement: Supplementary file 1 [file 12864_2026_13088_MOESM2_ESM.docx]

**Additional File 2: Fig S1-S6**

**Mild oxidative stress and dietary epigenetic modulators direct DNA methylation remodeling toward stress-resilience pathways**

Javier A. Rodriguez-Casariego^1^, Carmen Navarro-Guillén^2^, Rubén Huesa-Cerdán^2^, Juan Antonio Hidalgo-Perez^2^, Gonzalo Martínez-Rodríguez^2^, Erick Perera^2^*

^1^Institute of Environment, Florida International University, Miami, Florida, USA

^2^Department of Marine Biology and Aquaculture, Andalusian Institute of Marine Sciences (ICMAN), Spanish National Research Council (CSIC), 11519 Puerto Real, Cadiz, Spain

* Correspondence: [erick.perera@csic.es](mailto:erick.perera@csic.es)

**Supplementary Figure S1**: WGCNA network topology diagnostics. Analysis of soft-thresholding power selection for signed network construction using biweight midcorrelation. (A) Scale-free fit index (R^2) as a function of the soft-thresholding power; a power of 13 (black) was selected to achieve a scale-free topology fit R^2 > 0.85. (B) Mean connectivity as a function of the soft-thresholding power.

**Supplementary Figure S2:** Integration of pathway-level activity with WGCNA module eigengenes. Dotplot illustrating the correlation between sample-wise enrichment scores (ES) and module eigengenes (ME) for the Red, Turquoise, and Blue modules. Pathway scores were calculated using the ssGSEA method within the GSVA R package, utilizing Gene Ontology (GO) gene sets mapped to zebrafish orthologs. Displayed terms were restricted to those achieving a global GSEA significance of q < 0.05 and an absolute Normalized Enrichment Score (NES) > 1.8, with arrows indicating the direction of treatment effect (Up/Down). Point size represents the absolute Pearson correlation coefficient (|r|) between the ES and ME, while color indicates the statistical significance (-log10 FDR) of the association.

**Supplementary Figure S3**. General characterization of DNA methylation patterns in gilthead seabream liver. DNA methylation levels (average beta) in and around TSS and DNA methylation levels (average beta) in the context of CpG islands across the genome. Below: Relationship between DNA methylation and gene expression across genomic regions: Gene Body + Promoter (-2 kb to +200 bp from TSS), Promoter only, Gene body only.

**Supplementary Figure S4**: Frequency of Differentially methylated regions (DMR) overlapping genomic regions.

**Supplementary Figure S5**: Heatmap of DNA methylation levels (β) per sample for DMC enriched promoters (only promoters with > 5 DMCs).

**Supplementary Figure S6**: Heatmap of DNA methylation levels (β) per sample for DMC enriched extended genes (gene body + promoter with > 5 DMCs). Genes highlighted in red were also differentially expressed (DEGs).
